# BosR and PlzA reciprocally regulate RpoS function to sustain *Borrelia burgdorferi* in ticks and mammals

**DOI:** 10.1172/JCI166710

**Published:** 2023-03-01

**Authors:** André A. Grassmann, Rafal Tokarz, Caroline Golino, Melissa A. McLain, Ashley M. Groshong, Justin D. Radolf, Melissa J. Caimano

**Affiliations:** 1 Department of Medicine, UConn Health, Farmington, Connecticut, USA.; 2 Center for Infection and Immunity and; 3 Department of Epidemiology, Mailman School of Public Health, Columbia University, New York, New York, USA.; 4 Department of Pediatrics,; 5 Department of Molecular Biology and Biophysics,; 6 Department of Genetics and Genome Sciences, and; 7 Department of Immunology, UConn Health, Farmington, Connecticut, USA.

**Keywords:** Infectious disease, Microbiology, Bacterial infections, Molecular biology, Signal transduction

## Abstract

The RNA polymerase alternative σ factor RpoS in *Borrelia burgdorferi* (*Bb*), the Lyme disease pathogen, is responsible for programmatic-positive and -negative gene regulation essential for the spirochete’s dual-host enzootic cycle. RpoS is expressed during tick-to-mammal transmission and throughout mammalian infection. Although the mammalian-phase RpoS regulon is well described, its counterpart during the transmission blood meal is unknown. Here, we used *Bb*-specific transcript enrichment by tick-borne disease capture sequencing (TBDCapSeq) to compare the transcriptomes of WT and Δ*rpoS Bb* in engorged nymphs and following mammalian host-adaptation within dialysis membrane chambers. TBDCapSeq revealed dramatic changes in the contours of the RpoS regulon within ticks and mammals and further confirmed that RpoS-mediated repression is specific to the mammalian-phase of *Bb*’s enzootic cycle. We also provide evidence that RpoS-dependent gene regulation, including repression of tick-phase genes, is required for persistence in mice. Comparative transcriptomics of engineered *Bb* strains revealed that the *Borrelia* oxidative stress response regulator (BosR), a noncanonical Fur family member, and the cyclic diguanosine monophosphate (c-di-GMP) effector PlzA reciprocally regulate the function of RNA polymerase complexed with RpoS. BosR is required for RpoS-mediated transcription activation and repression in addition to its well-defined role promoting transcription of *rpoS* by the RNA polymerase alternative σ factor RpoN. During transmission, ligand-bound PlzA antagonizes RpoS-mediated repression, presumably acting through BosR.

## Introduction

Lyme disease (LD) is a multisystem infectious disorder caused by the highly motile, invasive spirochetal pathogen *Borrelia* *burgdorferi* (*Bb*) (1). With an estimated 476,000 cases diagnosed and treated annually, LD is easily the most prevalent arthropod-borne infection in the United States (2). In nature, *Bb* cycles between an *Ixodes* species vector and a vertebrate reservoir host, usually a small rodent; in North America, it is primarily the white-footed mouse (3, 4). The generalist feeding behavior of *Ixodes* species is responsible for transmission of *B*. *burgdorferi* to humans by infected ticks (1, 5). In recent years, much has been learned about the global regulatory systems that enable LD spirochetes to transit between their arthropod vector and mammalian reservoir host (6, 7). However, while there is ample evidence for crosstalk between pathways (7), the mechanisms by which they modulate each other’s regulatory output to create the appropriate transcriptomic and proteomic profile at a given point in the enzootic cycle remain obscure.

The RNA polymerase alternative σ factor RpoN/RpoS regulatory pathway, first described in a seminal report by Norgard and colleagues (8), controls gene expression via the effector alternative σ factor RpoS. RpoN, the LD spirochete’s sole other alternative σ factor (9), transcribes *rpoS* in response to environmental cues provided by the blood meal (6, 7); the pathway remains on throughout tick transmission and mammalian infection but rapidly turns off during larval acquisition (10–12). Spirochetes lacking RpoS are avirulent when introduced into mice by needle inoculation (6) and remain confined to the midgut during feeding when they are artificially introduced into ticks by immersion (13). The response regulatory protein 2 (Rrp2) and *Borrelia* oxidative stress response regulator (BosR), a ferric uptake regulator (fur) ortholog, are essential for transcription of *rpoS* in vitro and in vivo, presumably forming a complex with RNA polymerase–RpoN (RNAP-RpoN) holoenzyme (7). Comparison of the transcriptomes of *Bb* cultivated in vitro at 37°C and following mammalian host–adapted in dialysis membrane chambers (DMCs) (14) have brought to light 2 salient differences between the in vitro and in vivo RpoS regulons (11, 15). First, not all genes transcribed by RpoS in mammals are transcribed by RpoS in vitro, and, second, RpoS represses a subset of tick-phase genes upon mammalian host-adaptation but not in vitro. These differences imply that promoter recognition by RpoS differs in vitro and within mammals. The ability of RpoS to reciprocally regulate tick- and mammalian host–phase genes throughout the enzootic cycle led to its designation as the gatekeeper (15).

A second global regulatory system in *Bb* involves the ubiquitous bacterial second messenger bis-(3′-5′)-cyclic dimeric guanosine monophosphate (c-di-GMP) and the sensory transduction histidine kinase 1/response regulatory protein 1 (Hk1/Rrp1) two-component system (TCS) (7, 16). Binding of unidentified, exogenous ligand(s) generated within the midgut of feeding ticks to the periplasmic sensor domain of Hk1 initiates a signal-transduction cascade that culminates in phosphorylation of Rrp1 and synthesis of c-di-GMP (7, 16). Spirochetes lacking either Hk1 or Rrp1 host-adapt normally within DMCs and are virulent in mice but are destroyed within feeding ticks (10, 17–19). Thus, in contrast to the RpoN/RpoS pathway, the Hk1/Rrp1 pathway is tick-specific. Production of c-di-GMP by *Bb* results in the upregulation of genes involved in the utilization of alternative carbon sources and genes encoding cell envelope constituents required to defend against noxious substances and environmental stressors generated by the blood meal (20–22). Importantly, many tick-phase genes upregulated by c-di-GMP are repressed by RpoS within mammals (11). Efforts to elucidate c-di-GMP signaling in *Bb* have centered about PlzA, the sole PilZ domain protein in most LD spirochetes, including the B31 strain (7, 16, 23). In feeding ticks, deletion of *plzA* or complementation of Δ*plzA* with a PlzA protein unable to bind c-di-GMP, phenocopies deletion of *hk1* and *rrp1* (19, 24, 25). Our recent studies with the DMC system revealed that ectopic constitutive synthesis of c-di-GMP by *cDGC Bb*, acting through ligand-bound PlzA, exerts a ‘brake’ effect on RpoS-dependent gene regulation, antagonizing RpoS-mediated repression and reducing expression of some RpoS-upregulated genes (25). We interpreted these results to indicate that ligand-bound PlzA is a principal driver of RNAP-RpoS function in ticks and that transition to the mammalian host-phase RpoS regulon requires cessation of c-di-GMP synthesis.

Deconvolution of the processes shaping the RpoS regulon requires transcriptional profiling of WT and Δ*rpoS* spirochetes in feeding nymphs as well as in mammals. While DMCs provide sufficient host-adapted spirochetes for genome-wide transcriptomics, conventional RNA-Seq of *Bb* in feeding nymphs is not feasible due to an abundance of mouse and tick RNA. We circumvented this problem using probe-based enrichment prior to RNA-Seq to compare the WT and Δ*rpoS* transcriptomes in engorged nymphs and DMCs. These analyses revealed that the RpoS regulon changed dramatically as spirochetes transited from tick to mammal, with RpoS-mediated repression occurring strictly within mammals. RNA-Seq analysis of the *cDGC* strain in DMCs revealed that ligand-bound PlzA skewed the RpoS regulon toward a ‘tick-phase’ transcriptional profile. Using a Δ*rpoS* strain that expressed *rpoS* from an IPTG-inducible promoter (Δ*rpoS/irpoS*), we determined that persistence in mammals involved RpoS-upregulated genes as well as RpoS-mediated repression. Inactivation of *bosR* in Δ*rpoS*/*irpoS* abrogated RpoS-mediated repression and diminished RpoS-upregulation in DMCs. Thus, BosR was required not only for RpoN-dependent transcription of *rpoS* but also for downstream RpoS-dependent facets of host-adaptation. Remarkably, ectopic expression of RpoS in a Δ*bosR*Δ*rpoS/irpoS* background phenocopied the ‘brake effect’ of ligand-bound PlzA on RpoS-mediated repression in DMCs. Collectively, these results enabled us to formulate a working model whereby ligand-bound PlzA counteracted BosR during transmission to antagonize RpoS-mediated repression of tick-phase genes and diminish expression of RpoS-upregulated genes. Cessation of c-di-GMP synthesis with consequent release of PlzA-dependent antagonism following transmission reset RNAP-RpoS to its default position, which was maintained throughout mammalian infection.

## Results

### Development of capture-based enrichment RNA-Seq to delineate RpoS-regulated genes in engorged nymphal ticks

**Figure 1:**
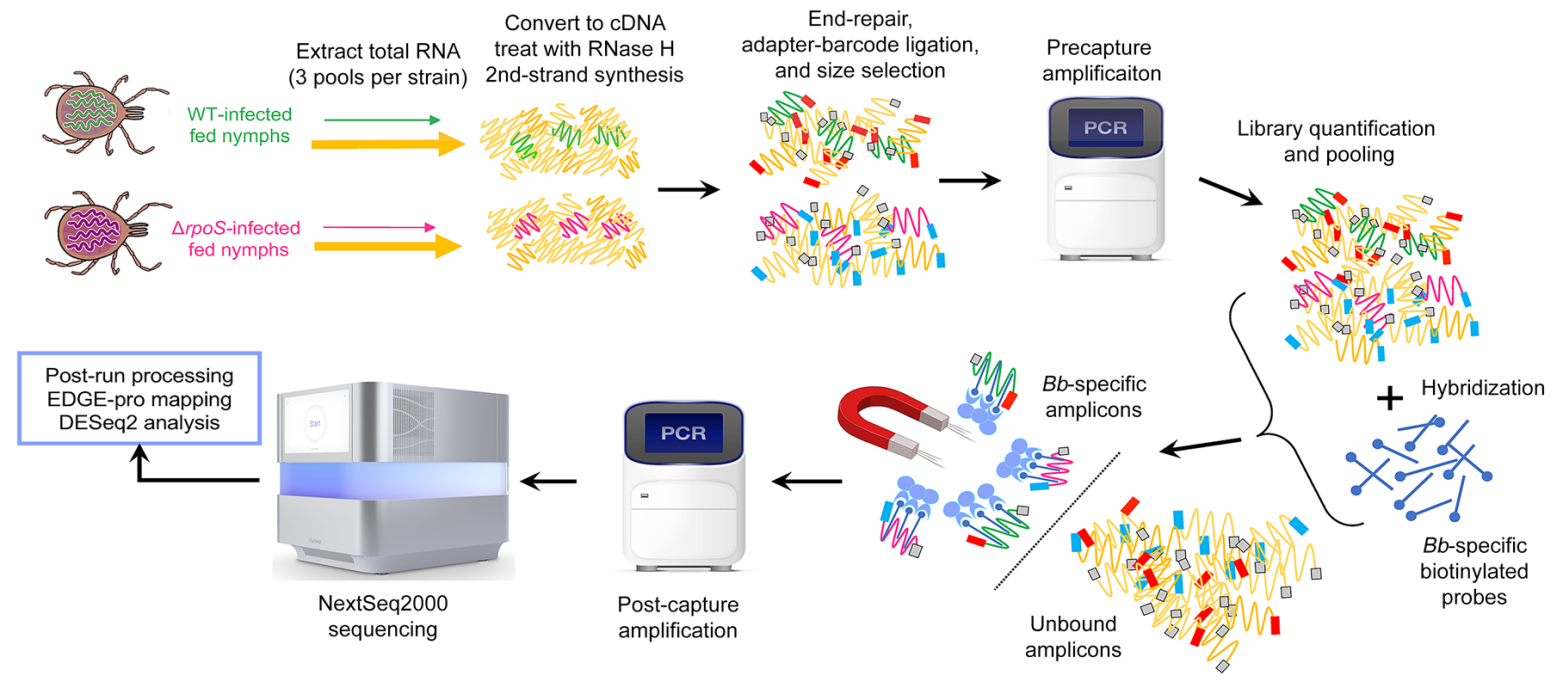
Workflow for TBDCapSeq. Total RNA extracted from fed nymphs infected with either WT (green) or Δ*rpoS* (magenta) *Bb* was converted to cDNA and used as input for second-strand synthesis. Libraries were prepared using dual-indexes (blue and red). Following precapture amplification, libraries were hybridized to *Bb*-specific biotinylated probes. *Bb*-specific amplicon–probe duplexes were captured using magnetic streptavidin beads (lilac), amplified using Illumina universal primers, and sequenced on a NextSeq2000. Raw reads were mapped using EDGE-pro and analyzed for differential gene expression using DESeq2. TBDCapSeq for DMC-cultivated samples was performed using the same pipeline.

Using comparative microarray and RNA-Seq, we previously defined the *Bb* RpoS regulon following temperature-shift in vitro and cultivation within DMCs (11, 15). Collectively, these studies demonstrated that mammalian host signals modulate promoter recognition by RNAP-RpoS and license RpoS-mediated repression of tick-phase genes. Notably, these studies identified a cohort of genes upregulated by RNAP-RpoS only in mammals. Given that RpoS is essential for transmission (13), we reasoned that the RpoS regulon includes genes upregulated exclusively during the nymphal blood meal. In a pilot RNA-Seq study using ribodepleted RNA from engorged nymphs infected with WT strain B31, only approximately 6,700 reads mapped to protein coding genes (0.034% of approximately 20 million total raw reads) (Supplemental Table 1; supplemental material available online with this article; https://doi.org/10.1172/JCI166710DS1), a value too low to obtain comprehensive transcriptomic data. To overcome this bottleneck, we took advantage of an enrichment strategy, designated TBDCapSeq, developed by Tokarz and colleagues (26, 27), which uses hybridization probes to ‘capture’ pathogen-specific amplicons prior to sequencing (Figure 1). Using TBDCapSeq, we compared the transcriptomes of WT and Δ*rpoS* *Bb* in fed nymphs and DMCs. Summaries of the raw and mapped data are presented in Supplemental Table 1.

### Overview of TBDCapSeq analyses

**Figure 2:**
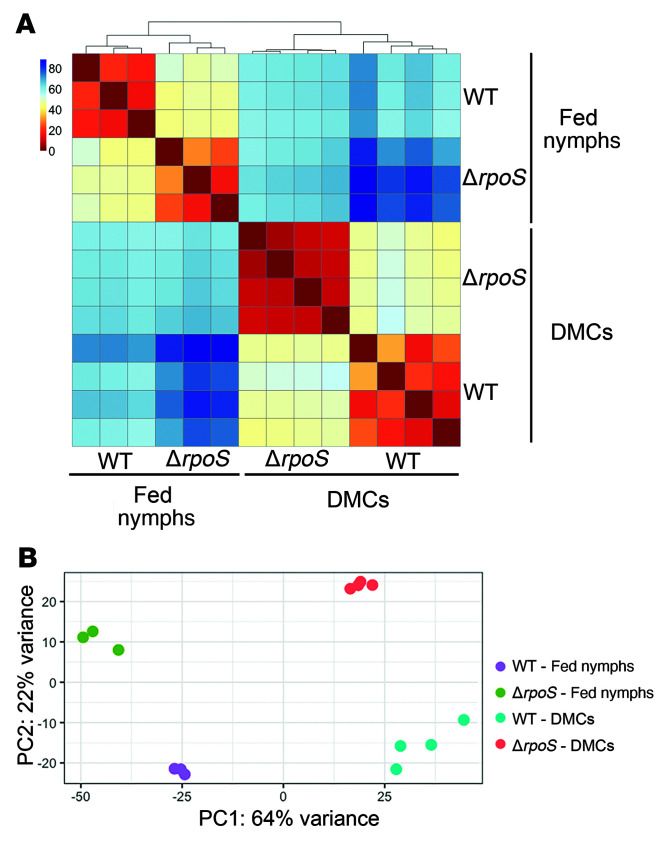
The contour of the *Bb* transcriptome varies substantially across the feeding nymphal tick and mammalian host phases of the enzootic cycle. Hierarchical clustering (**A**) and PCA plots (**B**) for WT and Δ*rpoS* *Bb* in fed nymphs (3 biological replicates per strain) and following cultivation in DMCs (4 biological replicates per strain) were generated using *R* Studio.

Approximately 11.3 and 15.6 million raw reads were obtained from fed nymphs infected with WT and Δ*rpoS* strains, respectively. Of these, approximately 30% were *Bb*-specific, representing an approximately 1,000-fold enrichment over conventional RNA-Seq. After post-run processing, approximately 1.6 and 1.9 million reads for protein coding genes in WT and Δ*rpoS*, respectively, remained. Of the 1,227 protein coding genes used for mapping, roughly 1,000 were detected at more than 10 transcripts per kilobase million (TPMs) in all 3 biological replicates (Supplemental Table 2). We obtained even more robust data for DMC-cultivated spirochetes. Of the approximately 44 million total reads obtained for WT and approximately 35 million total reads obtained for Δ*rpoS* DMC samples, roughly 21 and 17 million were *Bb*-specific, with 79 and 73% mapping to protein coding genes, respectively. Approximately 1,200 genes were detected at at least 10 TPMs in all 4 biological replicates (Supplemental Table 2). Prior microarray analyses demonstrated extensive transcriptomic remodeling as spirochetes transit between ticks and mammals (28). Along these lines, hierarchical clustering and Principal Component Analysis (PCA) plots (Figure 2) showed wide separation of WT transcriptomes in fed nymphs and mammals. The distance between WT and Δ*rpoS* suggests that RpoS is a major contributor to this transcriptional divergence. Indeed, DESeq2 identified 213 genes differentially regulated by RpoS in fed nymphs and/or DMCs. Of the 170 RpoS-regulated genes identified in DMCs, all but 3 (*bb0228*, *bb0454*, and *bbb29/malX-2*) were restored to near-WT levels by *trans*-complementation with *rpoS* expressed under its native promoter (Supplemental Table 3). To ascertain the extent of bias introduced by enrichment, we compared the RpoS DMC regulons obtained by TBDCapSeq and conventional RNA-Seq (11). Of the 98 RpoS-regulated genes identified in DMCs by conventional RNA-Seq, 89 — 55 upregulated and 34 repressed — were similarly regulated by TBDCapSeq (Supplemental Table 3). The high degree of overlap between these independent data sets minimized concerns that enrichment faithfully represents the spirochete transcriptome in a given milieu.

### The RpoS regulon changes dramatically when LD spirochetes transits from ticks to mammals

Genome-wide comparisons of WT and Δ*rpoS* *Bb* in fed nymphs and DMCs revealed that the RpoS regulon varies substantially across the enzootic cycle (7, 13). Of note, all key components of the RpoN/RpoS pathway (*bb0647*/*bosR*, *bb0763*/*rrp2,* *bb0450*/*rpoN* and *bb0771*/*rpoS*) were expressed at comparable levels in fed nymphs and DMCs (Supplemental Table 4), arguing against fluctuations in RpoS protein levels being responsible for these differences. 4 categories of differentially expressed genes were identified: (a) core genes upregulated by RpoS in both nymphs and DMCs; (b) genes upregulated by RpoS only in nymphs; (c) genes upregulated by RpoS only in DMCs; and (d) genes repressed by RpoS in mammals. Notably, no genes were repressed by RpoS during tick feeding.

#### Genes upregulated by RpoS in fed nymphs and DMCs.

In both fed nymphs and DMCs, 52 genes were upregulated by RpoS (hereafter designated core genes) (Supplemental Table 5). Eleven, including the RpoS-upregulated prototypes *bbb19/ospC* and *bba24*/*dbpA*, are known to be transcribed exclusively by RpoS (i.e., absolutely RpoS-dependent) in vitro and/or in DMCs (11, 13, 15, 29). Based on a comparison of TPM values for WT and Δ*rpoS* samples (Supplemental Table 2), 27 additional core genes also are considered absolutely RpoS-dependent. Twenty-two of the 38 absolutely RpoS-dependent core genes, most notably *ospC*, *dbpA* and *bbi42*, were transcribed at comparable levels in fed nymphs and DMCs. Thirteen, including 3 Pfam54_60 paralogs (*bba65*, *bba66* and *bba73*), the OspF paralog *bbo39* (*erpL*), and 2 Mlps (*bbp28/mlpA* and *bbm28*/*mlpF*), were transcribed at higher levels in fed nymphs, while 18 were higher in DMCs. The DMC-enhanced group included *vlsE1*, the expression site for the Vls system for antigenic variation (30), *bba34*/*oppA5*, encoding an oligopeptide substrate binding protein (11, 31), and *bbk32*, encoding a vascular endothelial adhesin and inhibitor of the classical complement pathway (32–34). Seven core genes, including 5 related to chemotaxis (*bb0680/mcp4*, *bb0681/mcp5*, *bb0671/cheX*, *bb0567/cheA-1,* and *bb0565/cheW-2*), were transcribed at appreciable levels by Δ *rpoS* *Bb*, indicating dual transcription by RpoS and RpoD. An additional 6 core genes (*bb0400*, *bb0798*, *bbi42*, *bbj27*, *bbk53,* and *bbq03*), all encoding hypothetical proteins, were dually transcribed by RpoS and RpoD only in DMCs.

#### Genes upregulated by RpoS only during tick transmission.

(Supplemental Table 6). Forty-four genes were designated tick-only genes because they were upregulated by RpoS only in feeding nymphs and not in DMCs. Of the 44, 40 were transcribed exclusively by RpoS in fed nymphs, while the remaining 4 (*bb0418*/*dipA*, *bb0637/nhaC1, bb0729*/*gltP*, and *bbh09*) were dually transcribed by RpoS and RpoD with a significant contribution to their expression from the former σ factor. In contrast, in DMCs, all 44 were either transcribed exclusively by RpoD or dually transcribed, but the contribution of RpoS to their expression was not statistically significant. Thus, the σ factor selectivity for genes in this group differs between ticks and mammals, with significant upregulation by RpoS occurring only in fed nymphs (≥ 3-fold difference with *q* ≤ 0.05). Nine tick-only genes, including the Pfam54_60 paralogs *bba64* and *bbe31*, are required for transmission (35–38).

#### Genes upregulated by RpoS only within mammals.

(Supplemental Table 7). Forty genes were upregulated by RpoS only within DMCs. Unlike the tick-only genes, which were transcribed to varying extents in ticks and mammals, the vast majority of DMC-only RpoS-upregulated genes were expressed exclusively in mammals (Supplemental Table 2). Two-thirds, 67%, of the DMC-only genes appeared to be absolutely RpoS-dependent, including 17 encoded on lp28-2; the contribution of this linear plasmid to virulence has not been established (11, 39). The remaining 13 DMC-only genes, including 5 related to motility and chemotaxis (*bb0273/fliR*, *bb0578/mcp-1*, *bb0669/cheA-2*, and *bb0670/cheW-3*), were dually transcribed by RpoS and RpoD in mammals.

#### Genes repressed by RpoS within mammals.

(Supplemental Table 8). Seventy-seven RpoS-regulated genes were expressed at significantly lower levels in WT versus Δ*rpoS* in DMCs and, hence, are repressed by RpoS “(≥ 3-fold difference with *q* ≤ 0.05). RpoS-repressed genes fell into 2 groups. The first consisted of genes that were expressed at comparable levels by WT and Δ*rpoS Bb* in fed nymphs but were strongly repressed by RpoS in DMCs. Twenty of these tick-phase genes, including *bba15*/*ospA*, *bba16/ospB*, *bba62/lp6.6*, *bba68/BbCRASP1*, and the *glp* operon (*bb0240-0243*), were shown previously to be repressed by RpoS in mammals (11, 15, 25). TBDCapSeq also identified an additional 10 RpoS-repressed genes in this group, including *bb0330*/*oppA3*, encoding an oligopeptide substrate binding protein (31), and *bba69*, encoding a Pfam54_60 lipoprotein (35). The remaining 47 RpoS-repressed genes were transcribed by WT *Bb* at comparably low levels in feeding nymphs and DMCs, but showed increased expression in the absence of RpoS in DMCs. This second category of RpoS-repressed genes included 3 closely related Pfam54_60 paralogs (*bbi36*, *bbi38*, and *bbi39*) (35) and *bbd18*, encoding a known regulator of RpoS protein levels (40, 41). Given its importance to the RpoS pathway, we confirmed the expression profile for *bbd18* by qRT-PCR. *bbd18* was transcribed at virtually identical low levels in fed nymphs and DMCs but was upregulated 10-fold in DMC-cultivated Δ*rpoS* *Bb* (Supplemental Figure 1). Presumably, RpoS-mediated repression of *bbd18* in mammals ensured that levels of this regulatory protein remain low in mammals, when RpoS is essential.

### Genes differentially regulated in feeding nymphs and/or mammals independent of RpoS

A dividend of TBDCapSeq is that it enables assessment of the RpoS-independent as well as the RpoS-dependent components of the *Bb* transcriptome in ticks and mammals (Supplemental Table 9). Examination of hierarchical clustering and PCA plots for Δ*rpoS* in fed nymphs and DMCs (Figure 2) suggested that RpoS-independent, differentially expressed genes comprise a substantial component of the WT transcriptomes in these 2 milieus. After excluding RpoS-regulated genes, 250 genes differed by more than 3-fold (*q* ≤ 0.05) between feeding nymphs and DMCs (Supplemental Table 9). Seventy-five genes were expressed at higher levels in fed nymphs, while 175 were higher in DMCs. Most of the RpoS-independent genes upregulated in feeding nymphs encode proteins with housekeeping functions — i.e., DNA replication, cell division, and protein translation and turnover — or functions related to nutrient acquisition and intermediary metabolism. Utilization of alternate carbon sources is critical to spirochete fitness in ticks (42, 43). Five genes (*bb0166*/*malQ*, *bb0367*, *bb0557*/*ptsH-2*, *bb0559*/*crr*, and *bb0629*/*fruA-2*) encode components of the phosphoenolpyruvate-dependent sugar phosphotransferase system — the spirochete’s central pathway for carbohydrate transport (42, 43) — and could be involved in uptake of alternative carbon sources. Eight are related to cell wall biosynthesis, including the chitobiose transporter *bbb04-06/chbCAB*, as well as *bb0151/nagA*, *bb0201/murE*, and *bb0841/arcA*. Increased expression of *chb* is particularly noteworthy given that chitobiose can be used for energy generation as well as cell wall biosynthesis (20, 44). Finally, 3 encode putative regulatory proteins — BB0355, a CarD-like transcriptional regulator required for transmission (45); BB0785/SpoVG, a tick-phase DNA/RNA-binding protein of undetermined function (46, 47); and BB0047/BpuR, a DNA/RNA-binding protein — were upregulated in feeding ticks (48) (Supplemental Table 4). Of the 75 RpoS-independent genes, 6 (*bb0166*/*malQ*, *spoVG, bbb04-06/chbCAB*, and *bbb07*) expressed at higher levels in fed nymphs are upregulated by c-di-GMP in vitro (10).

While a large majority (78%) of RpoS-independent genes upregulated in DMCs encode hypothetical proteins, 14 encode gene products related to DNA replication (*bb0455*, *bb0552/ligA*, and *bb0632/recD*), influx/efflux of small molecules (*bb0642/potA* and *bb0641/potB*), biosynthesis of metabolic cofactors (*bb0782/nadD* and *bb0589/pta*), purine salvage (*bb0384/bmpC*, *bb0467*, *bb0524,* and *bbb23*), and maintenance of the cell envelope (*bb0304*/*murF*, *bb0586/femA*, and *bb0721/pgsA*) (49–54). Also noteworthy, *bb0733*/*plzA*, which has a virulence-related function in mice unrelated to binding of c-di-GMP (23–25), was upregulated in DMCs compared with fed nymphs. With the exception of *rrp1*, which was slightly higher in mammals, all other known or putative regulatory factors (7) were expressed at comparable levels in both milieus (Supplemental Table 4).

### Ligand-bound PlzA impairs RpoS-mediated repression and diminishes transcription of some RpoS-upregulated genes

**Figure 3:**
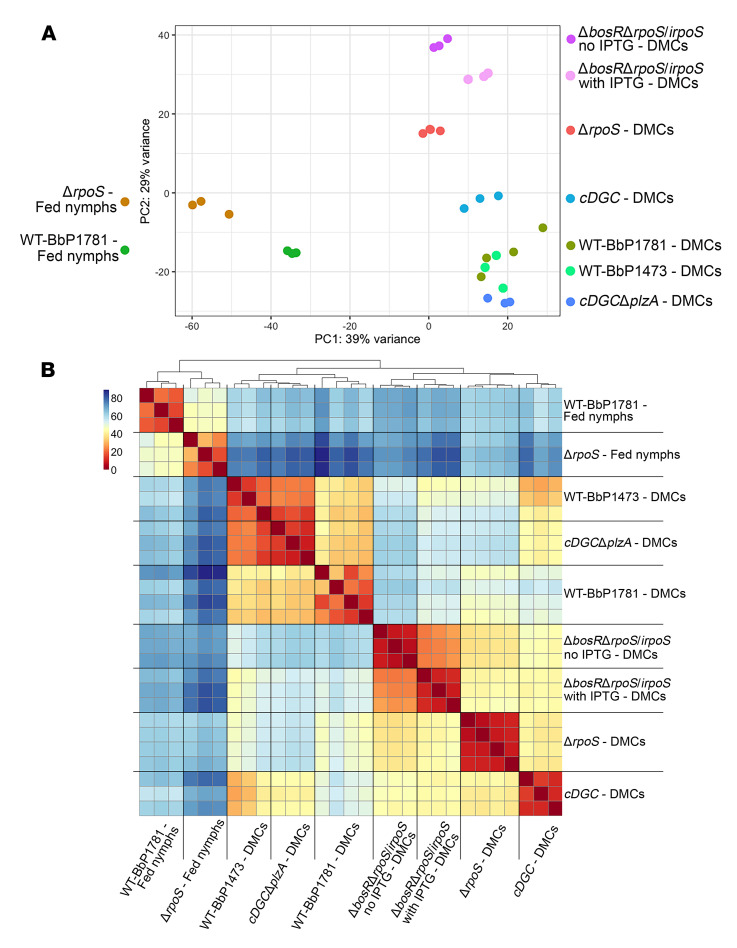
Interplay between RpoS, BosR, and ligand-bound PlzA regulates differential gene expression in feeding nymphal ticks and mammals. PCA (**A**) and hierarchical clustering (**B**) for (i) DMC-cultivated isogenic WT (WT-BbP1781), Δ*rpoS*, and Δ*bosR*Δ*rpoS/irpoS* with and without IPTG; (ii) DMC-cultivated isogenic WT (WT-BbP1473), *cDGC*, and *cDGC*Δ*plzA*; and (iii) isogenic WT (WT-BbP1781) and Δ*rpoS* within fed nymphs.

Using a strain, *cDGC*, that constitutively synthesizes c-di-GMP in mammals, we previously demonstrated that ligand-bound PlzA acts as a ‘brake’ on RpoS-dependent gene regulation, antagonizing RpoS-mediated repression and diminishing expression of RpoS-upregulated genes (25). These data led us to propose that ligand-bound PlzA is a principal determinant of the RpoS regulon during transmission and, moreover, that transition to the mammalian host-phase RpoS regulon requires cessation of c-di-GMP synthesis. To garner support for this notion on a genome-wide scale, we performed TBDCapSeq on isogenic WT, *cDGC*, and *cDGC*Δ*plzA* strains cultivated in DMCs (Supplemental Tables 5–8). As noted previously (25), transcripts for *rpoS* were unaffected by either increased c-di-GMP or loss of PlzA (Supplemental Table 4). In contrast, ligand-bound PlzA had a striking effect on the RpoS regulon. Of the 77 genes repressed by RpoS in DMCs, 57, including 26 of the 30 tick-phase genes noted above, were expressed at significantly higher levels in *cDGC* compared with WT (≥ 3-fold difference with *q* ≤ 0.05). In every case, deletion of *plzA* restored RpoS-mediated repression in the *cDGC*Δ*plzA* strain. The modulatory effect of ligand-bound PlzA in mammals also extended to 17 RpoS-upregulated genes. Expression of 10 RpoS core genes, including *ospC*, *dbpA*, *bbk32*, and *vlsE1*, and 7 DMC-only genes decreased significantly in the *cDGC* strain compared with WT; all but 2 (*bb0580* and *bb0578/mcp-1*) were absolutely RpoS-dependent (≥ 3-fold difference with *q* ≤ 0.05). In all but 1 case, deletion of PlzA in the *cDGC* strain restored RpoS-upregulation to WT levels; *vlsE1*, the sole outlier, was transcribed at lower levels in the *cDGC* strain in a PlzA-independent manner (Supplemental Table 5). Expression of *vlsE1* also requires the trans-acting factor YebC (55). The negative effect of c-di-GMP on RpoS-upregulation of *vlsE1* raises the possibility that YebC is c-di-GMP-regulated through some unknown mechanism. Of note, 4 tick-only RpoS-upregulated genes (*bbh32*, *bbk01*, *erpA*, and *erpB*) were transcribed at higher levels by *cDGC* in a PlzA-dependent manner. A question that arose from the above data was whether ligand-bound PlzA acts predominantly on genes within the RpoS regulon. As illustrated by the PCA plot and hierarchical clustering (Figure 3), synthesis of c-di-GMP in mammals appears to shift the transcriptome of *cDGC* toward that of Δ*rpoS*, while *cDGC*Δ*plzA* clustered closely with WT (Figure 3A). Collectively, these data suggested that the modulatory effect of c-di-GMP on RNAP-RpoS was largely PlzA-dependent and that the influence of ligand-bound PlzA outside of the RpoS regulon was negligible.

### Persistence of *Bb* infection in mice requires RpoS and involves RpoS-mediated repression of tick-phase genes

**Figure 4:**
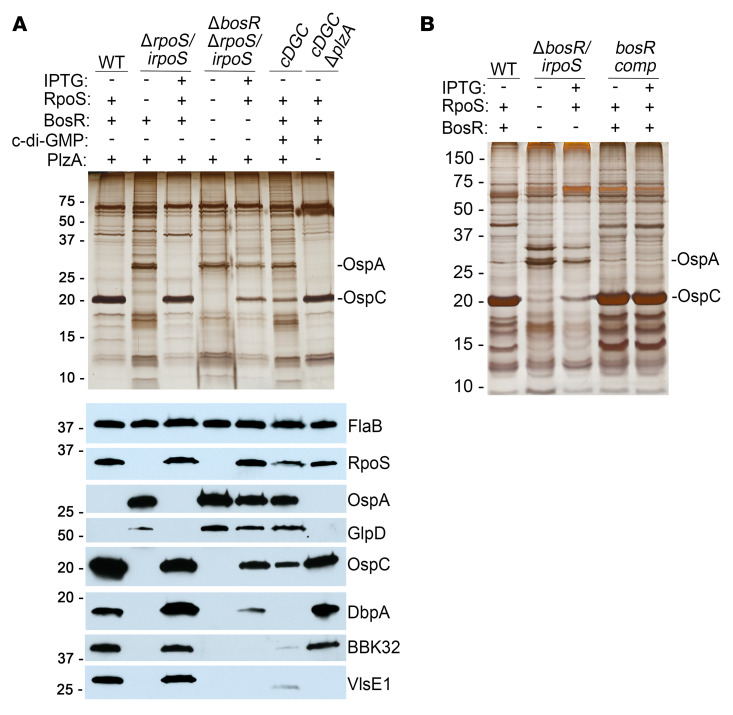
Ligand-bound PlzA and BosR modulate the RpoS regulon in a reciprocal manner within mammals. (**A**) Lysates from DMC-cultivated WT, Δ*rpoS/irpoS,* Δ*bosR*Δ*rpoS/irpoS*, *cDGC,* and *cDGC*Δ*plzA* were separated by SDS-PAGE and stained with silver or immunoblotted with antisera against FlaB, RpoS, OspA, GlpD, OspC, DbpA, BBK32, and VlsE. (**B**) Lysates from DMC-cultivated WT, Δ*bosR/irpoS,* and *bosRcomp/irpoS* were separated by SDS-PAGE and stained with silver. Molecular weight markers (kDa) are shown at the left of each gel. “+” and “–” indicate the presence or absence of IPTG, RpoS, BosR, and PlzA, and/or c-di-GMP synthesis by the constitutively active diguanylate cyclase in *cDGC* strains. **A** and **B** show representative images from 3 biological replicates per strain. Uncropped immunoblots for Figure 4A are provided in Supplemental Figure 5.

Using a Δ*rpoS* strain complemented in trans (11), we previously demonstrated that loss of the complementing plasmid placed spirochetes at a survival disadvantage for up to 20 weeks following needle inoculation, supporting a requirement for RpoS during persistent infection. These studies also suggested that RpoS-mediated repression is maintained throughout infection. To confirm the requirement for RpoS-upregulated genes and RpoS-mediated repression for persistence, we developed a Δ*rpoS* strain (Δ*rpoS/irpoS*) harboring an IPTG-inducible copy of the *rpoS* gene inserted into the highly stable endogenous cp26 plasmid (25). When cultivated in vitro, Δ*rpoS/irpoS* expressed RpoS and prototypical RpoS-upregulated gene products in an IPTG concentration–dependent manner (Supplemental Figure 2A). As previously reported (56), over-expression of *rpoS* (i.e., more than 50 μM IPTG) was toxic (Supplemental Figure 2B). To determine whether physiological levels of RpoS could be induced in Δ*rpoS/irpoS* within animals, we implanted DMCs containing Δ*rpoS/irpoS* into rats receiving IPTG in their drinking water. Oral administration of IPTG yielded levels of RpoS and RpoS-upregulated proteins and repression of OspA and GlpD at levels comparable to those of DMC-cultivated WT *Bb* (Figure 4A). By immunoblot, we also confirmed the RpoS-dependence of *vlsE1* revealed by RNA-Seq (Figure 4A).

**Figure 5:**
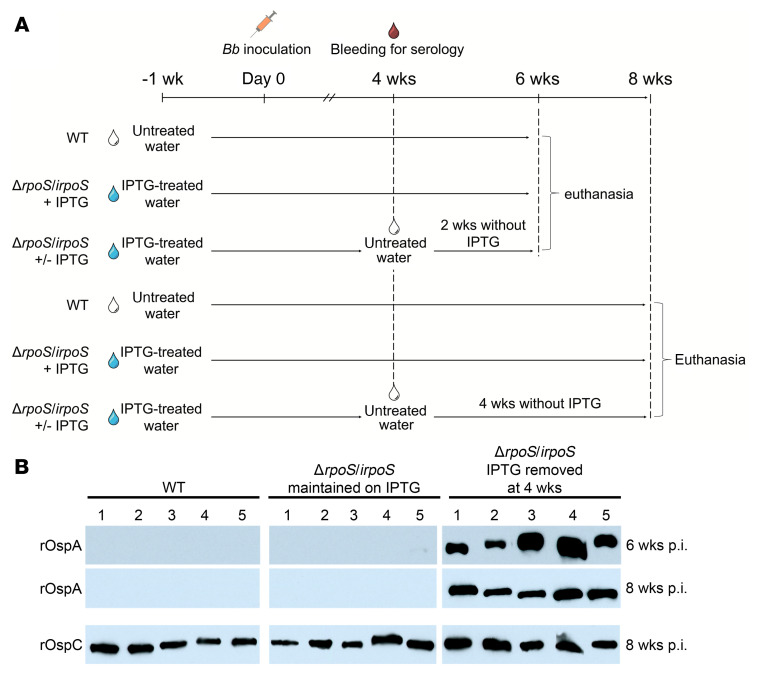
RpoS is required for persistence in mice. (**A**) Experimental design to assess the contribution of RpoS to persistence in C3H/HeJ and SCID mice (5 mice per condition, per time point). Mice infected with Δ*rpoS/irpoS* received IPTG-treated water (blue) 1 week before inoculation. Serology was performed 4 weeks after inoculation to confirm infection (Supplemental Figure 3). At 4 weeks, IPTG was withdrawn from half of the Δ*rpoS/irpoS*-infected mice, while the other half received IPTG for the remainder of the experiment. WT-infected mice received untreated water (white) throughout the experiment. At 6 and 8 weeks after inoculation (p.i.), mice were euthanized for collection of blood for serology and tissues for culture (Table 2). (**B**) Loss of RpoS was associated with production of antibodies against OspA. Sera from individual C3H/HeJ mice collected at 6 and 8 weeks after inoculation was assayed by immunoblot using 100 ng of recombinant OspA. Sera collected 8 weeks after infection was also assayed against 100 ng of recombinant OspC. Uncropped immunoblots for Figure 5B are provided in Supplemental Figure 6.

Having established that Δ*rpoS/irpoS* *Bb* host-adapts normally in rats given IPTG, we used this strain to assess the contribution of RpoS to persistence in mice. First, we confirmed the infectivity of Δ*rpoS/irpoS* by inoculating C3H/HeJ mice. As shown in Table 1, nearly all tissues from mice infected with either WT — which received untreated water — or Δ*rpoS/irpoS* — which received IPTG-treated water — were culture-positive 2-weeks after inoculation, while untreated mice infected with Δ*rpoS/irpoS* were culture-negative. Tilly and colleagues (57, 58) previously established that OspC is dispensable for infectivity by approximately 21 days after inoculation. To avoid an OspC-related phenotype in our persistence experiments, C3H/HeJ mice infected with Δ*rpoS/irpoS* were maintained on IPTG-treated water for at least 4 weeks after inoculation (Figure 5A). At the 4-week time point, IPTG was removed from half of the Δ*rpoS/irpoS*-infected mice, while the other half was maintained on IPTG-treated water. At 6 and 8 weeks after inoculation, WT- and Δ*rpoS/irpoS-*infected mice maintained on IPTG were culture positive from most tissues (Table 2). However, 2 weeks after stopping IPTG-treatment (6 weeks after inoculation), only 4 of 30 tissues from Δ*rpoS/irpoS*-infected mice were culture positive, with a single positive site per animal. All tissues from Δ*rpoS/irpoS*-infected mice were culture negative 4 weeks after discontinuation of IPTG treatment. Antibodies against OspC were detected in sera from all mice 8 weeks after inoculation (Figure 5B). Strikingly, Δ*rpoS/irpoS*-infected mice mounted strong anti-OspA responses after discontinuation of IPTG-treatment, whereas OspA antibodies were not detected in Δ*rpoS/irpoS*-infected mice continuing to receive IPTG (Figure 5B).

To investigate whether antibodies were responsible for clearance of Δ*rpoS/irpoS* following withdrawal of IPTG, we repeated the above experiment using NOD.Cg-Prkdc^SCID^/J (SCID) mice. As with C3H/HeJ mice, SCID mice inoculated with Δ*rpoS/irpoS* and maintained on IPTG-treated water for the entire experiment, as well as mice infected with WT *Bb*, were culture positive 6 and 8 weeks after inoculation (Table 2). Two weeks after discontinuation of IPTG treatment, Δ*rpoS/irpoS* spirochetes were recovered from 9 of 30 tissue sites cultured. 4 weeks after removal of IPTG, only 3 of 30 sites from Δ*rpoS/irpoS-*infected mice were culture positive. Collectively, these data demonstrate that the requirement for RpoS extends beyond early infection and that RpoS-dependent factors functionally unrelated to adaptive immunity are also required to sustain infection.

### BosR is essential for transcriptional as well as repressive functions of RpoS

In addition to serving as an activator for RpoN-dependent transcription of *rpoS*, BosR also has been proposed as a repressor for *ospA* and other tick-phase genes (59, 60). The latter studies, however, were conducted in vitro and failed to divorce the requirement of BosR for RpoN-dependent transcription of *rpoS* from its putative repressor function. We reasoned that our IPTG-inducible *irpoS* allele, which dissociates transcription of *rpoS* from the Rrp2/BosR/RpoN complex, could be used to clarify the contribution of BosR to RpoS-mediated repression. Accordingly, we inactivated *bosR* in Δ*rpoS/irpoS*, generating the strain Δ*bosR*Δ*rpoS/irpoS*. During in vitro cultivation without IPTG, Δ*bosR*Δ*rpoS/irpoS* expressed neither RpoS nor OspC, whereas both were expressed in a dose-dependent manner when IPTG was added to the culture medium (Supplemental Figure 2C). Surprisingly, deletion of *bosR* ameliorated RpoS toxicity at IPTG concentrations above 50 μM (Supplemental Figure 2D). Although Δ*bosR*Δ*rpoS/irpoS Bb* cultivated in DMCs in IPTG-treated rats expressed WT levels of RpoS, we observed noticeably lower levels of OspC, DbpA, BBK32, and VlsE along with incomplete repression of OspA and GlpD; this protein profile was strikingly similar to that of DMC-cultivated *cDGC* (Figure 4A). Complementation of Δ*bosR*Δ*rpoS/irpoS* was technically challenging due to the paucity of antibiotic-resistance markers available for selection in *Bb*. As an alternative, we generated a *bosR/irpoS* strain that retained the native *rpoS* gene. Like Δ*bosR*Δ*rpoS/irpoS*, Δ*bosR/irpoS* grew normally in vitro in the presence of over 50 μM IPTG (Supplemental Figure 2E) and showed dysregulation of RNAP-RpoS function when cultivated in DMCs in IPTG-treated rats (Figure 4B). Complementation of *bosR* in the Δ*bosR/irpoS* background (*bosRcomp*) restored RpoS-mediated toxicity during in vitro cultivation with more than 50 μM IPTG (Supplemental Figure 2F) as well as RpoS-dependent facets of mammalian host-adaption in rats given IPTG (Figure 4B). Moreover, unlike Δ*rpoS/irpoS*, Δ*bosR*Δ*rpoS/irpoS* was avirulent in C3H/HeJ and SCID mice treated with IPTG (Table 3), demonstrating that murine infectivity requires BosR as well as RpoS.

We next performed RNA-Seq on DMC-cultivated Δ*bosR*Δ*rpoS/irpoS* *Bb* with and without IPTG to determine the RpoN-independent contribution of BosR to shaping the RpoS regulon in mammals (Supplemental Table 3). Of the 92 RpoS-upregulated genes in DMCs — 52 core and 40 DMC-only — 53 required BosR for transcription, as they were not upregulated in the Δ*bosR*Δ*rpoS/irpoS* strain under inducing conditions (Supplemental Table 5 and 7). Moreover, all but 2 of the remaining 39 RpoS-upregulated genes showed lower folds of regulation in the absence of BosR. For example, transcripts for *ospC* increased by only 14-fold following induction of RpoS in Δ*bosR*Δ*rpoS/irpoS* compared with 984-fold in WT compared with Δ*rpoS* (Supplemental Tables 3 and 5). Indeed, the immunoblots for OspC, DbpA,and BBK32 revealed that these transcriptional differences appear to be biologically relevant at the protein level (Figure 4A). Most strikingly, 75 of 77 RpoS-repressed genes were not downregulated in Δ*bosR*Δ*rpoS/irpoS* despite induction of RpoS (Supplemental Table 8). The above results indicated that RNAP-RpoS function in mammals is highly dependent on BosR. This conclusion was supported by PCA and hierarchical clustering analyses (Figure 3), which suggest similarity between the transcriptomes of Δ*rpoS* and Δ*bosR*Δ*rpoS/irpoS* in IPTG-treated rats. In contrast, the effect of ligand-bound PlzA on RpoS-dependent gene regulation was selective, affecting only 55 of 75 BosR/RpoS-repressed genes (Supplemental Table 8) and 16 of 90 BosR/RpoS-upregulated genes in DMCs (Supplemental Tables 5 and 7).

## Discussion

The ability of microorganisms to adapt rapidly and reversibly to endogenous and exogenous signals is essential for survival in dynamic, often hostile, environments. Consequently, most bacteria have evolved a general stress response to defend against initiating threats as well as seemingly unrelated stresses (61, 62). In *E*. *coli* and other γ-proteobacteria these broad adaptive responses are coordinated by the alternative σ factor σ^s^/RpoS (61, 62). The strict dual host lifestyle of *Bb*, on the other hand, presents LD spirochetes with predictable exogenous and endogenous signals that have enabled them to develop programmatic transcriptional responses for each phase of the enzootic cycle (7, 16). The requirement for c-di-GMP, acting primarily through PlzA, during tick feeding has established the importance of this second messenger for vector adaptation (7, 16). *Bb* also has appropriated an RpoS distantly related to its Gram-negative prototype to regulate a parallel adaptive response required for migration out of the nymphal midgut, but that, unlike c-di-GMP signaling, continues following transmission (7, 11). TBDCapSeq revealed that the RpoS-ON state during transmission and mammalian infection produces distinct transcriptional profiles based on the presence or absence of c-di-GMP, respectively. These results mirror recent findings demonstrating that the c-di-GMP effector PlzA toggles between tick- and mammalian-phase conformations based on c-di-GMP binding (11, 63). Functional overlap between these evolutionarily related σ factors RpoS and RpoD is well-recognized in other bacteria (64–67). Herein we show that BosR and ligand-bound PlzA function in a reciprocal manner to contour the RpoS regulon in ticks and mammals by modulating promoter recognition by RNAP-RpoS and RNAP-RpoD.

**Figure 6:**
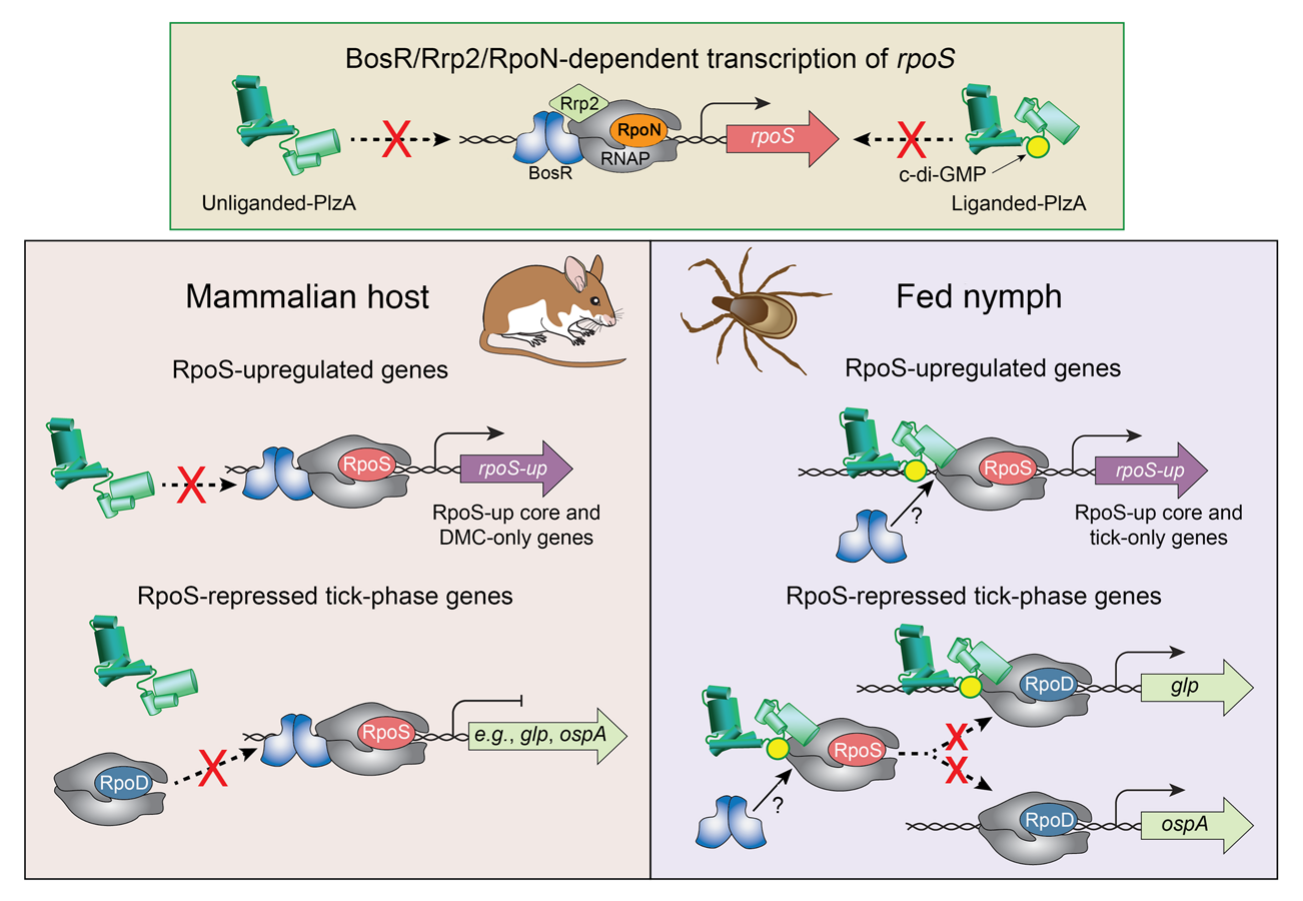
Proposed model for the reciprocal regulation of the RpoS gatekeeper by BosR and ligand-bound PlzA. Top: Transcription of *rpoS* by RNAP complexed with BosR/Rrp2/RpoN in feeding nymphs and mammals is unaffected by either c-di-GMP (yellow circle) or PlzA. Left: In mammals, BosR enhances transcription of RpoS-upregulated core and DMC-only genes and is required for RpoS-mediated repression of tick-phase genes. RNAP-RpoS/BosR complex binds upstream of RpoS-repressed tick-phase genes, including *ospA* and the *glp* operon, preventing transcription by RNAP-RpoD. Due to the absence of c-di-GMP within mammals, *apo* PlzA is unable to interact with RNAP and/or prevent BosR’s σ activator function. Right: In feeding nymphs, ligand-bound PlzA interferes with BosR function, reducing expression of some RpoS-upregulated genes, including *ospC, dbpA,* and *vlsE*, and antagonizing RpoS-mediated repression either by blocking BosR binding to RNAP-RpoS or allosteric interactions with RNAP-RpoS/BosR. Based on this model, BosR’s σ activator function is specific to RNAP-RpoS, while ligand-bound PlzA interacts with both RNAP-RpoS and RNAP-RpoD in feeding nymphs. Ligand-bound PlzA also is required for RpoD-dependent transcription of *glp* genes, while tick-phase genes with strong promoters, such as *ospA*, are transcribed by RNAP-RpoD alone.

It is universally accepted that *Bb*’s Fur ortholog BosR forms a complex with RNAP-RpoN and the response regulator Rrp2 to transcribe *rpoS* (7) (Figure 6). Whether BosR serves additional transcriptional role(s) has been a matter of debate. Seshu, Hyde, and colleagues (68, 69) reported that BosR activates an oxidative stress response in vitro following exposure to *t*-butyl peroxide. By TBDCapSeq, however, we saw no differences in transcript levels for putative BosR-dependent genes (i.e., *napA* and *sod*) associated with detoxification of ROS in ticks or DMCs. We note that our study was not designed to identify putative BosR-dependent, RpoS-independent genes. Shi, et al. (60) found that when expressed at supra-physiological levels in vitro in a Δ*rpoS* strain, BosR binds to *cis* sites upstream of the *ospA* promoter to block transcription by RNAP-RpoD. Our current and previous studies showed clearly that RpoS-mediated repression of tick-phase genes, including *ospA*, is a mammalian host–phase phenomenon that does not occur in the absence of RpoS (11, 15, 70). Our experiments with a *Bb* strain that expresses an IPTG-inducible *rpoS* in a Δ*bosR* background resolved these ostensibly discordant findings in an unexpected manner; RNAP-RpoS was unable to downregulate tick-phase genes without BosR, implying that repression requires a collaboration between the two. Moreover, collaboration between BosR and RNAP-RpoS extends beyond repression of prototypical tick-phase genes. TBDCapSeq revealed a second group of BosR/RpoS-repressed genes, exemplified by *bbd18*, that are transcribed exclusively by RNAP-RpoD in feeding nymphs and DMCs (Supplemental Table 2). In the absence of RpoS, however, transcript levels for these genes are significantly increased only in mammals. We interpret these data to mean that the promoters for these genes are recognized by RNAP-RpoD more efficiently in mammals and that RpoS-mediated repression is required to ensure basal levels of expression during infection. Negative regulation by competing σ factors (i.e., promoter occlusion) is well-recognized in other bacteria, including *E*. *coli* (71, 72). In the case of *bbd18*, derepression following acquisition by a naive vector, when RpoN-dependent transcription of *rpoS* is off, likely enhances degradation of residual RpoS to facilitate midgut colonization (41). Remarkably, BosR also was required for optimal expression of many RpoS-upregulated genes in DMCs, indicating that it functions as a transcriptional activator for RpoS as well as for RpoN.

Canonical Furs repress transcription by metal-dependent binding to DNA at one or more conserved palindromic sites, or fur boxes, thereby blocking promoter recognition by RNAP-RpoD (73, 74). Ouyang, et al. (75) previously identified BosR boxes upstream of *rpoS*. However, only a handful of RpoS-regulated genes identified by TBDCapSeq contain putative BosR boxes within their upstream regions (75); thus, it seems unlikely that DNA binding by BosR is a prerequisite for all of its modulatory functions. In other bacteria, factors designated σ activators regulate promoter recognition by RNAPs, including RNAP-RpoS, without binding to specific DNA sequences (76, 77). In *E*. *coli*, the RpoS-specific σ activator Crl facilitates and stabilizes holoenzyme assembly by tethering RpoS to RNAP *via* the β′ subunit clamp toe domain (76, 78, 79). Although structurally unrelated to Crl, BosR could be acting analogously by recruiting RNAP to RpoS-dependent promoters. BosR is predicted to have noncanonical structural features that potentially explain its postulated ability to interact with DNA and RNAP-RpoS (Supplemental Figure 4). It contains an elongated, C-terminal intrinsically disordered region (IDR) reminiscent of another RpoS-specific σ activator, *Pseudomonas aeruginosa* SutA, whose C-terminal IDR stabilizes its interaction with RNAP (80, 81). Although BosR contains a highly conserved structural metal-binding site (*i.e.*, CxxC motif,), which is required for dimerization, it lacks a recognizable regulatory metal binding site (7). BosR also contains an additional α-helix within its N-terminal DNA-binding domain (82, 83). Previously, we mapped the RpoS repression site for *ospA* to within 47 nucleotides upstream of the transcriptional start site (70). Conceivably, DNA binding by BosR is particularly important for anchoring RNAP-RpoS holoenzyme at or near the promoters for tick-phase genes, blocking recognition by RNAP-RpoD. Along these same lines, the BosR-dependent toxicity associated with overexpression of RpoS in vitro likely reflects overexpression of RpoS-upregulated genes and/or reduced expression of one or more essential gene products due to competition with RNAP-RpoD (84).

The mechanism by which ligand-bound PlzA serves as the effector for c-di-GMP-dependent survival in ticks remains unclear. *Klebsiella pneumoniae* MrkH, a c-di-GMP-dependent transcriptional activator and PlzA ortholog (25, 85), provides a structural framework for deconvoluting PlzA’s global regulatory functions. Binding of c-di-GMP by MrkH induces conformational changes that enable it to bind to DNA and the C-terminal domain of RNAP α subunit (α-CTD) (85). As with MrkH, c-di-GMP binding by PlzA brings together its N-terminal PilZN3 and C-terminal PilZ β-barrels and likely positions 3 positively charged helices within the PilZN3 domain to create a potential interface for DNA binding (25, 63, 86). Based on an analysis of PlzA-dependent expression of *glpF*, the prototypical c-di-GMP-regulated, tick-phase gene, Zhang et al. (87) proposed that PlzA interacts directly with RNAP-RpoD. Our prior and current results are in accord with this supposition (10, 25). Tan et al. (85) identified 5 surface-exposed residues on the α-CTD required for MrkH-dependent transcription of the *mrkHI* operon; all 5 residues (L271, R276, N279, C280, and E284) are conserved in *Bb* α-CTD. Unlike MrkH, PlzA also modulates RNAP-RpoS function, an activity, which, to our knowledge, has not been described for other c-di-GMP-dependent transactivators. The reciprocal effects of ligand-bound PlzA and BosR, observed for *cDGC* and Δ*bosR*Δ*rpoS/irpoS*, at the transcriptional and protein levels, is compelling evidence that ligand-bound PlzA exerts its brake effect on RNAP-RpoS *via* BosR. This supposition leads to 2 possible scenarios (Figure 6). One is that ligand-bound PlzA prevents BosR from interacting with RNAP-RpoS or displaces RpoS from the RNAP holoenzyme complex. The other is that BosR remains bound to RNAP-RpoS, but ligand-bound PlzA negates BosR’s transactivator effect on RNAP-RpoS. Regardless of the mechanism, ligand-bound PlzA must be viewed as a major driving force for shaping the RpoS regulon during transmission, preventing repression of tick-phase genes and fine-tuning RpoS-dependent upregulation. In parallel, release of RpoS-mediated repression enables ligand-bound PlzA to positively regulate expression of a subset of tick-phase genes, such as *glp*s (10, 88), while transcription of other tick-phase genes, such as *ospA*, by RNAP-RpoD, is PlzA-independent (10, 25). That transcription of *rpoS* by the BosR/Rrp2/RpoN complex is unaffected by ligand-bound PlzA (Figure 6) underscores the specificity of these postulated PlzA-BosR interactions for RNAP-RpoS complexed with both BosR and RpoS. As important as ligand-bound PlzA is for modulating the RpoS regulon during transmission, the wide divergence between *cDGC* in DMCs and WT *Bb* in feeding nymphs points to substantial input from RpoS-independent regulatory factors, including the 3 (SpoVG, BpuR, and CarD) identified by TBDCapSeq, in shaping the global *Bb* transcriptome in ticks.

During transmission, the RpoS-ON state is transient, remaining active in ticks only during feeding (approximately 96 hours after attachment) or perhaps shortly thereafter during the postrepletion period. Not so, however, in mammals. After establishing themselves at the site of inoculation, LD spirochetes must not only disseminate but also persist at metastatic cutaneous sites within a reservoir-competent host long enough to be acquired by a naive ixodid vector. Previously, Ouyang et al. (12) showed that *rpoS* transcripts could be detected in chronically infected mice but did not examine whether survival of spirochetes during chronic infection depends upon continuance of RpoS-dependent gene regulation. Use of an IPTG-inducible *rpoS* allele (*irpoS*) confirmed that RpoS is absolutely required for persistence in mice. Moreover, the appearance of OspA antibodies is compelling evidence that continued expression of RpoS also sustains the repression of tick-phase genes. Clearance of Δ*rpoS/irpoS* spirochetes, however, was not immediate. The mammalian host–phase regulon provides multiple, mutually nonexclusive explanations for the delayed killing of organisms deprived of RpoS. Spirochetes unable to downregulate OspA cannot survive in mice (89). One, therefore, is that derepression of tick-phase lipoproteins elicits a protective antibody response. Spirochetes lacking the *vls* locus or unable to undergo recombinatorial switching are markedly attenuated in immunocompetent mice (30). Only recently has it become apparent that transcription of *vlsE,* the expression site for variable Vls lipoproteins, is RpoS-dependent (11). Parenthetically, since expression of *vlsE* requires the YebC transcription factor (55), this result implies that YebC collaborates with RNAP-RpoS. Loss of BBK32 would render spirochetes sensitive to antibody-mediated killing by the classical complement pathway, compounding the effects of antibody production to dysregulated tick-phase proteins and loss of VlsE defenses (32, 33). Infectivity data for Δ*rpoS/irpoS* in SCID mice argue that factors unrelated to adaptive immunity also contribute to clearance. The RpoS DMC regulon encodes multiple gene products involved in nutrient acquisition (e.g., OppA5), evasion of complement-mediated killing (e.g., OspEs), and chemotaxis (11, 90–93).

Comprehensive understanding of how RpoS sustains persistence will require interrogation of individual RpoS-regulated gene products throughout the mammalian host phase. Once acquired by a naive tick, rapid reversion to the RpoS-OFF state (11–13) enables unconstrained expression of tick-phase genes. While we now possess considerable insights into the mechanisms that regulate the contours of the RpoS regulon, we have none into the underlying phenomenon of how *Bb* distinguishes between the acquisition and transmission blood meals to determine whether RpoS should be on or off.

## Methods

### Cultivation of bacterial strains.

Bacterial strains and plasmids used in these studies are described in Supplemental Tables 10 and 12, respectively. Details regarding routine cultivation of *E*. *coli* and *Bb* in vitro and in DMCs are provided in Supplemental Methods.

### Routine DNA manipulation and cloning.

Oligonucleotide primers used in these studies (Supplemental Table 11) were purchased from Sigma-Aldrich. Routine cloning was performed by In-Fusion HD Cloning (TaKaRa Bio Inc.). Routine and high-fidelity PCR amplifications were performed using RedTaq (Denville Scientific) and CloneAmp HiFi (TaKaRa Bio Inc.), respectively. *Bb* strains were transformed by electroporation (94). Details regarding generation of *Bb* strains expressing an IPTG-inducible *rpoS* allele and IPTG-induction are described in Supplemental Methods.

### Murine and tick infection studies.

Female C3H/HeJ or NOD.Cg-*Prkd*^SCID^/J (SCID) mice (The Jackson Laboratory) were inoculated with 1 × 10^5^ organisms via intradermal injection. 4-to-8 weeks after inoculation, animals were sacrificed, and blood and tissues were collected for serology and culturing, respectively. Pathogen-free *Ixodes scapularis* larvae were purchased from Oklahoma State University Tick Rearing Facility (Stillwater, Oklahoma, USA). Naive larvae were infected by immersion (95), fed to repletion on naive C3H/HeJ mice, and allowed to molt. Infected nymphs were fed on naive C3H/HeJ mice until fully engorged as previously described (13, 96).

### SDS-PAGE and immunoblotting.

Details regarding routine SDS-PAGE and immunoblotting of *Bb* are provided in Supplemental Methods. Polyclonal antisera against FlaB (97), OspC (11), DbpA (98), GlpD (99), RpoS (100), and OspA (11) were previously described. Antisera against BBK32 C1/C1r domain (33) and VlsE C6 peptide (101) were generated by immunizing Sprague-Dawley rats (Envigo RMS Inc.) with the corresponding purified, recombinant His-tagged protein, as previously described (102).

### RNA-Seq.

Detailed methods for TBDCapSeq and conventional RNA-Seq are provided in Supplemental Methods. A schematic overview of TBDCapSeq is presented in Figure 1. Total RNA was isolated from engorged nymphs or DMCs, as previously described (11), converted to cDNA using SuperScript IV (Thermo Fisher Scientific), treated with RNase H, followed by second-strand synthesis with Klenow fragment (New England Biolabs). Libraries were prepared with the KAPA Hyperplus kit (Roche) using 25–50 ng of input material, according to manufacturer’s instructions. Amplified libraries were quantified, equalized, and pooled. A total of 1 μg of library pool was mixed with 5 μg of COT human DNA (Thermo Fisher Scientific) and 2 nmol of blocking oligo pool (Roche) and then dehydrated. To enrich for *Bb*-specific transcripts, the dried pool was resuspended in 7.5 μL Hybridization Buffer and 3 μL Hybridization Component A (Roche) and heated at 95°C for 5 minutes before the addition of 4.5 μL of custom biotinylated TBD SeqCap EZ Probes (26, 27). The mixture was heated at 95°C for 5 min and incubated at 47°C for 16–20 h. After incubation, the probes were pulled down using magnetic streptavidin SeqCap Capture beads (Roche) and washed with buffers of decreasing stringency (SeqCap EZ Hybridization and Wash Kit; Roche). The *Bb*-enriched material was then amplified for 16 cycles using Illumina universal primers with KAPA HiFi HotStart Ready Mix (Roche), quantified on a TapeStation 4200 (Agilent Technologies), and sequenced on a NextSeq2000 (Illumina) that generated 150 nucleotide single-end reads. Raw read data for conventional and TBDCapSeq were processed, mapped, and analyzed as described in Supplemental Methods. Raw data have been deposited in the NCBI Sequence Read Archive (SRA) database (PRJNA881286; Supplemental Table 1).

### Statistics.

Pairwise quantitative reverse-transcriptase PCR comparisons were evaluated by unpaired 2-tailed Student’s *t* tests with a 95% confidence interval using Prism v8.4.3 (GraphPad). A *P* value of less than 0.05 was considered statistically significant. Differential gene expression was calculated for RNA-Seq data sets using DESeq2 (103). Genes that differed by at least 3-fold with a FDR-adjusted *P* value (*q* value) of 0.05 or under were considered differentially expressed.

### Study approval.

All experiments involving animals were approved by the UConn Health IACUC.

## Author contributions

MJC, AAG, AMG, JDR, and RT conceptualized the project. MJC, AAG, CG, AMG, MAM, and RT performed experiments. MJC, AAG, and JDR performed the analyses. MJC, AAG, AMG, GO, and RT developed the methodology. MJC, AAG, and JDR supervised the project. MJC, AAG, and JDR wrote the original draft of the manuscript. MJC, AAG, AMG, JDR, and RT reviewed and edited the manuscript.

## Supplementary Material

Supplemental data

Supplemental table 2

Supplemental table 3

Supplemental table 4

Supplemental table 9

## Figures and Tables

**Table 1:**
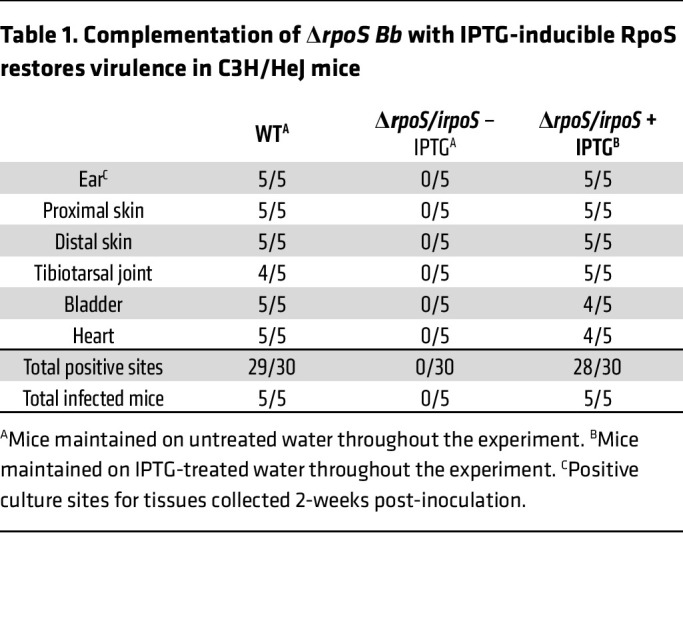
Complementation of Δ*rpoS* *Bb* with IPTG-inducible RpoS restores virulence in C3H/HeJ mice

**Table 2:**
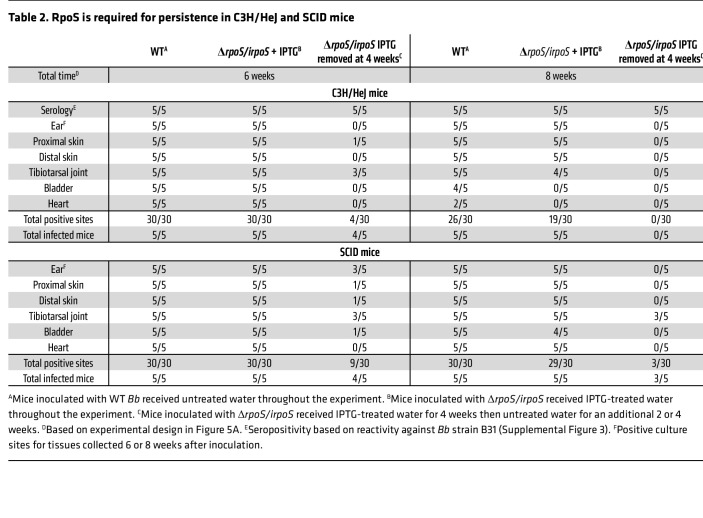
RpoS is required for persistence in C3H/HeJ and SCID mice

**Table 3:**
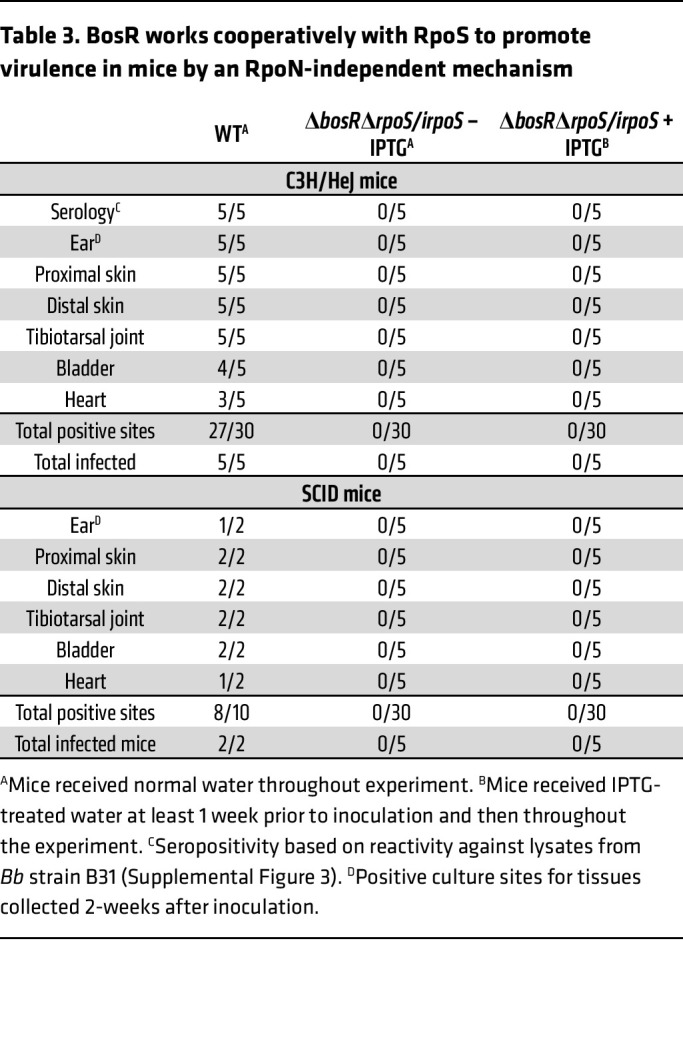
BosR works cooperatively with RpoS to promote virulence in mice by an RpoN-independent mechanism

## Version 1 01/17/2023

In-Press Preview

## Version 2 03/01/2023

Electronic publication
