# Supplementary material for: BosR and PlzA reciprocally regulate RpoS function to sustain Borrelia burgdorferi in ticks and mammals
Source: J Clin Invest. 2023 Mar 1;133(5):e166710. doi: 10.1172/JCI166710 (PMC9974103; doi:10.1172/JCI166710)
Supplement: Supplemental data [file jci-133-166710-s113.pdf]

Supplemental Material

BosR and PlzA reciprocally regulate RpoS function to sustain *Borrelia burgdorferi* in ticks and mammals

André A. Grassmann[1], Rafal Tokarz[2,3], Caroline Golino[1], Melissa A. McLain[1],

Ashley M. Groshong[1,4,‡], Justin D. Radolf[1,4-7*] and Melissa J. Caimano[1,4,5*]

Departments of [1]Medicine, [4]Pediatrics, [5]Molecular Biology and Biophysics, [6]Genetics and Genome Sciences, and [7]Immunology, UConn Health, Farmington, CT, 06030, USA

[2]Center for Infection and Immunity and [3]Department of Epidemiology, Mailman School of Public Health, Columbia University, New York City, NY, 10032, USA

[‡] Current address: Laboratory of Bacteriology, Rocky Mountain Laboratories, Division of Intramural Research, National Institute of Allergy and Infectious Diseases, National Institutes of Health, Hamilton, MT, 59840, USA

*Corresponding authors

Justin D. Radolf, MD
263 Farmington Avenue
Farmington, CT 06030-3715
jradolf@uchc.edu
Tel: 860-679-8480

Melissa J. Caimano, PhD
263 Farmington Avenue
Farmington, CT 06030-3715
mcaima@uchc.edu
Tel: 860-679-7312

Supplemental Methods

Cultivation of bacterial strains

Escherichia coli strains Top10 (ThermoFisher Scientific, Waltham, MA) and Stellar (TaKaRa Bio USA, Inc., San Jose, CA), used for cloning and isolation of plasmid DNA, were maintained in Lysogeny broth (LB) or LB agar supplemented with the appropriate antibiotics (ampicillin, 100 µg/ml; spectinomycin, 100 µg/ml; kanamycin, 100 µg/ml; and/or gentamicin, 5 µg/ml). *B. burgdorferi* strains (Supplemental Table 10) were maintained in Barbour-Stoenner-Kelly (BSK)-II medium (1) supplemented with 6% rabbit serum (Pel-Freeze Biologicals, Rogers, AR) with the addition of *Borrelia* antibiotic cocktail (kanamycin, 400 µg/ml; streptomycin, 100 µg/ml; gentamicin, 50 µg/ml) when appropriate; plasmid contents of *B. burgdorferi* strains used in these studies were monitored as previously described (2). For standard growth curves, *B. burgdorferi* cultures were inoculated at a starting density of 1×10^4 spirochetes/ml in BSK-II containing the appropriate antibiotics and cultivated at 37˚C for up to 10 days. Spirochetes were enumerated daily by darkfield microscopy using a Petroff-Hausser counting chamber (Hausser Scientific Co., Horsham, PA). *Bb* strains (1×10^4 *Bb*/ml starting density) were cultivated in the peritoneal cavities of Sprague-Dawley rats (either sex; Envigo RMS, Inc., Indianapolis, IN) within dialysis membrane chambers (DMCs) for 12-14 days as previously described (3, 4). Tissues harvested from infected mice were cultured at 37˚C in BSK-II medium containing *Borrelia* antibiotic cocktail (0.05 mg/ml sulfamethoxazole, 0.02 mg/ml phosphomycin, 0.05 mg/ml rifampicin, 0.01 mg/ml trimethoprim and 2.5 µg/ml amphotericin B).

Routine DNA manipulation and cloning

Plasmids were purified from *E. coli* using QIAprep spin, midi or mega kits (Qiagen, Germantown, MD) or NucleoBond PC2000 (TaKaRa Bio USA, Inc.). Bacterial genomic DNA was extracted using the Gentra Puregene Yeast/Bacteria kit (Qiagen). Oligonucleotide primers used in these studies (Supplemental Table 11) were purchased from Sigma-Aldrich (St. Louis, MO). Except where noted, cloning was performed using the In-Fusion HD Cloning Plus kit (TaKaRa Bio USA, Inc.). Routine and high-fidelity PCR amplifications were performed using RedTaq (Denville Scientific, Holliston, MA) and CloneAmp HiFi (TaKaRa Bio USA, Inc.), respectively. Sanger sequencing of cloned DNAs was performed by Genewiz, Inc. (South Plainfield, NJ) and analyzed using MacVector (MacVector, Inc., Apex, NC). *B. burgdorferi* strains were transformed by electroporation as previously described (5).

Generation of *B. burgdorferi irpoS* and *cDGC* strains. Supplemental Table 12 presents a complete list of bacterial plasmids used in these studies. To generate an IPTG-inducible *rpoS* (*irpoS*) gene, *bb0771/rpoS* was amplified from *Bb* strain B31 5A4 using primers *rpoS*-5' and -3' and then cloned into *Nde*I/*Hind*III-digested pJSB275 (6), replacing the luciferase gene. The pQE30-*rpoS*/P*flaB*-*lacI* region was PCR-amplified using primers *irpoS*-*lacI*-5' and 3' and cloned into a pUC19-based suicide vector for insertion into cp26 (7). The resulting plasmid (EcAG291) was confirmed by sequencing and then used to transform Δ*rpoS* (BbP1752) (8) and Δ*bosR* (OY10) (9), yielding Δ*rpoS/irpoS* (BbAG351) and Δ*bosR/irpoS* (BbAG580), respectively. To generate Δ*bosR*Δ*rpoS/irpoS* (BbAG646), a fragment encoding *bb0647/bosR* plus ~1-kb of flanking DNA was amplified from strain B31 using primers *bosR*-5' and *bosR*-3'and cloned into *Bam*HI-digested pUC19, creating pUC19/*bosR*. This plasmid was linearized by inverse PCR using primers invpUC*bosR*-5' and-3', replacing *bosR* with the P*flgB*-*kanR* cassette, amplified from pBSV2 (10) using *bosR*-*kanR*-5' and -3'. The resulting plasmid (pMC5115) was transformed into Δ*rpoS/irpoS*. To complement *bosR*, the spectinomycin-resistance cassette (*PflgB*-*aadA*) from pJSB275 was amplified using

primers *bosR*compSS-5' and -3' then cloned downstream of the *bosR* coding region in pUC19/*bosR*, linearized by inverse PCR using the primers invpUC*bosR*-5' and invpUC*bosR*comp-3'. The resulting plasmid (pMC4925) was transformed into Δ*bosR/irpoS*, generating *bosRcomp/irpoS* (BbAG643). To generate a *Bb* strain (BbAG545; *cDGC*) expressing the constitutively active diguanylate cyclase Slr1143 from *Synechocystis* sp., WT B31 A3-68 (BbP1473) was transformed with the plasmid EcAG391 (2), containing P*flaB-slr1143-HA* flanked by ~1-kb of upstream and downstream sequence for *rrp1*. Genotypes for all *Bb* strains were confirmed by amplicon sequencing.

IPTG induction of *rpo*S in vitro and in vivo. For induction of *rpoS* in vitro, *irpoS* strains were grown in BSK-II containing the appropriate antibiotics and concentrations of IPTG ranging from 0.01 to 1.0 mM IPTG as previously described (2). For IPTG induction during DMC cultivation or murine infection (see below), normal (*i.e.*, untreated) drinking water was replaced with water containing 2% sucrose and 80 mM IPTG for at least seven days before DMC implantation or inoculation of mice and then throughout the duration of the experiment.

SDS-PAGE and immunoblotting. Whole-cell lysates prepared from *B. burgdorferi* strains cultivated to late logarithmic phase following temperature-shift to 37˚C were separated (~2 × 10^7 cells/lane) on 12.5% SDS-PAGE mini-gels and stained with silver as previously described (11). Polyclonal antisera against BB0147/FlaB (12), BBB19/OspC (8), BBA24/DbpA (13), BB0243/GlpD (14), BB0771/RpoS (15) and BBA15/OspA (8) were previously described. Recombinant BBK32 C1/C1r domain (16), generously provided by Dr. Brandon Garcia (East Carolina University, Greenville, NC), and VlsE C6 peptide (17), produced as previously described (18) were used to generate antisera by immunizing Sprague-Dawley rats with the corresponding recombinant His-tagged proteins using Freund's adjuvant (Sigma-Aldrich) as previously described (18). For immunoblotting, whole cell lysates were separated by SDS-PAGE, transferred to nitrocellulose, and incubated overnight with primary antibody (diluted 1:1000 – 1:15,000), followed by horseradish peroxidase (HRP)-conjugated goat anti-rat secondary antibody (Southern Biotechnology Associates, Birmingham, AL) diluted 1:30,000. Seroconversion in infectivity experiments (see below) was determined by immunoblotting *B. burgdorferi* strain B31 whole cell lysates (~2 ×10^7 cells per lane) with 1:1000 dilutions of sera from individual mice, followed by incubation with HRP-conjugated secondary antibody (Southern Biotechnology Associates) diluted 1:30,000. Immunoblots were developed using the Pierce SuperSignal West Pico chemiluminescence substrate (ThermoFisher Scientific).

Conventional RNAseq. Total RNA (3-4 biological replicates per strain) was isolated using TRIzol (ThermoFisher Scientific) from engorged nymphs (72-96 hrs post-placement) or following cultivation in DMCs with the designated *Bb* strains (Supplemental Table 1) and treated twice with TURBO DNA-*free* kit (ThermoFisher Scientific) followed by purification using RNeasy columns (Qiagen) as previously described (8). Samples were eluted in RNase-free water and purified RNAs were analyzed using Qubit RNA HS Assay Kit (ThermoFisher Scientific) and/or TapeStation 4200 (Agilent Technologies, Santa Clara, CA) using the RNA High Sensitivity assay. Libraries were prepared using Illumina Stranded Total RNA Ligation kit (Illumina, Inc., San Diego, CA), which includes ribodepletion, according to manufacturer's instructions. Libraries were validated for length and adapter dimer removal using the TapeStation 4200 D1000 high-sensitivity assay and then quantified and normalized using the double-stranded DNA (dsDNA) high-sensitivity assay for Qubit 3.0 (ThermoFisher Scientific). Libraries were run on a NovaSeq6000 (Illumina). Raw reads for each sample were trimmed using Sickle (v. 1.3.3) (19) and then mapped using

EDGE-pro version 1.1.3 (20) using custom fasta, protein translation table (ptt) and ribosomal/transfer RNA (rnt) files based on strain B31 (21, 22). In the custom fasta and ppt files, highly conserved (>90% identity) hypothetical genes encoded by cp32 plasmids are represented by plasmids cp32-1 (AE001575.1) and cp32-4 (AE001577.1), while genes encoding unique *mlp*, *ospE, ospF* and *elp* paralogs, and plasmid-specific partitioning regions (*pf32-pf49*) for the remaining seven cp32 plasmids are represented individually. Pseudogenes and genes encoding open reading frames <60 amino acids were excluded. Differential expression between strains and/or conditions was determined using DESeq2 (23). Principal component analysis (PCA) plots and hierarchical heatmaps were genesrated in R studio (24) using gplots, ggplot2, gtools and pheatmap packages (23). Raw read data have been deposited in the NCBI Sequence Read Archive (SRA) database (PRJNA881286; Supplemental Table 1).

Tick-borne Diseases Capture sequencing. A schematic overview of the TBDCapSeq workflow is presented in Figure 1. Total RNA was isolated from pools of 6-8 fully engorged nymphs (3 pools per strain) or DMCs (3-4 biological replicates per strain) using TRIzol and treated with DNase as previously described (8). DNA-free total RNA was converted to cDNA using SuperScript IV reverse transcriptase (ThermoFisher Scientific), treated with RNase H, followed by second-strand synthesis with Klenow fragment (New England Biolabs, Ipswich, MA). DNA concentrations were measured with the Qubit High Sensitivity Double-stranded DNA kit and Qubit 2.0 Fluorometer (ThermoFisher Scientific). Libraries with custom dual-indexes were prepared with the KAPA Hyperplus kit (Roche, Indianapolis, IN) using 25–50 ng of input material and the recommended adaptor concentrations and cycling parameters. Amplified libraries were quantified on a TapeStation 4200 using the D1000 kit (Agilent Technologies). Measured DNA concentrations were used to equalize libraries before pooling. After quantification on the TapeStation 4200, 1 μg of the pool was mixed with 5 μg of COT Human DNA (ThermoFisher Scientific) and 2000 pmol of Blocking Oligo pool (Roche). The mixture was fully dehydrated at 60°C in a vacuum centrifuge. To enrich for *Bb*-specific transcripts, the dried pool was resuspended in 7.5 μl Hybridization Buffer and 3 μl Hybridization Component A (Roche) and heated at 95°C for 5 min before the addition of 4.5 μl of custom biotinylated TBD SeqCap EZ Probe pool (Roche) containing overlapping biotinylated probes designed for strain B31 (SeqCap EZ Designs, v4.0; Roche) (14). The mixture was again heated at 95°C for 5 min before being incubated at 47°C for 16–20 h. After incubation, the probes were pulled down using magnetic streptavidin SeqCap Capture beads (Roche) and washed with buffers of decreasing stringency (SeqCap EZ Hybridization and Wash Kit, Roche). The *Borrelia*-enriched material was then amplified for 16 cycles using Illumina universal primers (Kapa HiFi HotStart Ready Mix; Roche). Finally, the amplified pool was quantified on a TapeStation 4200 and sequenced on a NextSeq2000 platform (Illumina) that generated 150 nt single-end reads. Raw read data were mapped and analyzed as described above. Raw reads were processed, mapped, and analyzed for differential gene expression as described above for conventional RNAseq. Transcripts per kilobase million (TPM) values were calculated as previously described (25) using reads mapped to borrelial protein coding sequences. Raw data have been deposited in the NCBI Sequence Read Archive (SRA) database (PRJNA881286; Supplemental Table 1).

qRT-PCR, Total RNA from engorged nymphs infected with WT *Bb* or DMC-cultivated WT and Δ*rpoS* organisms (3-6 biological replicate per condition, per strain) was isolated as described above. DNase-treated RNA was converted to cDNA using SuperScript III (ThermoFisher Scientific) and assayed in quadruplicate using SsoAdvanced Universal SYBR (*bbd18*) or Universal Probe (*flaB*) Mix (Bio-Rad, Hercules, CA) with primers described in Supplemental Table 11. Transcript copy numbers were calculated using the iCycler

post-run analysis software based on internal standard curves and then normalized against *flaB* as previously described (26).

Bioinformatics. Conserved domain searches were performed using Conserved Domain Database (CDD) Search (27), UniProt (28) and/or InterPro (29). Subcellular localization predictions were performed using BUSCA (Bologna Unified Subcellular Component Annotator) (30). Lipoprotein designations were based on Setubal *et al*. (31) and/or SignalP 6.0 (32). Outer membrane protein designations were based on Kenedy *et al*. (33). Multiple sequence alignments were generated by Clustal Omega (34) and MAFFT v. 7 (35). Structural modeling and intrinsically disordered region prediction for BosR were performed using AlphaFold (36, 37) and DISOPRED3 (38), respectively. PyMOL Molecular Graphics System v 2.3.2 (Schrödinger, LLC, New York, NY) was used for structure visualization and image rendering.

Supplemental Table 1. Summary of raw and mapped read data for all RNAseq analyses.

Strain #	Sample	SRA accession number	Objective/ Experiment	RNAseq Method	Total raw reads	Total processed reads	*Bb*-mapped reads	% *Bb* mapped reads	Reads mapped to *Bb* CDS	% CDS-mapped reads
1781	WT FedNym #1	SRR21604450	WT transcriptome in fed nymphs	Conventional	18,743,657	18,629,072	7,572	0.04	2,506	33.10
1781	WT FedNym #2	SRR21604449		Conventional	21,817,459	21,688,740	16,134	0.07	9,455	58.60
1781	WT FedNym #3	SRR21604434		Conventional	18,984,676	18,858,110	17,044	0.09	8,052	47.24
1781	WT FedNym #1	SRR21604424	RpoS regulon in fed nymphs; Fed nymphs vs. DMC comparison	TBDCapSeq	8,511,828	8,493,225	2,670,125	31.44	1,295,523	48.52
1781	WT FedNym #2	SRR21604440		TBDCapSeq	11,337,197	11,307,890	4,031,018	35.65	2,063,424	51.19
1781	WT FedNym #3	SRR21604418		TBDCapSeq	14,129,974	14,090,747	3,496,867	24.82	1,480,781	42.35
1752	Δ*rpoS* FedNym #1	SRR21604417	RpoS regulon in fed nymphs	TBDCapSeq	21,097,521	21,039,129	7,918,186	37.64	2,933,710	37.05
1752	Δ*rpoS* FedNym #2	SRR21604437		TBDCapSeq	9,235,548	9,203,752	2,525,706	27.44	927,691	36.73
1752	Δ*rpoS* FedNym #3	SRR21604416		TBDCapSeq	16,542,811	16,497,012	5,645,699	34.22	1,951,921	34.57
1781	WT DMC #1	SRR21604415	RpoS regulon in DMCs; Fed nymphs vs. DMC comparison	TBDCapSeq	36,049,348	36,017,248	17,871,632	49.62	13,961,761	78.12
1781	WT DMC #2	SRR21604448		TBDCapSeq	55,038,197	54,988,421	28,047,848	51.01	22,766,913	81.17
1781	WT DMC #3	SRR21604447		TBDCapSeq	35,510,757	35,478,963	16,847,998	47.49	12,148,858	72.11
1781	WT DMC #4	SRR21604445		TBDCapSeq	49,863,300	49,824,557	22,801,088	45.76	19,007,389	83.36
1752	Δ*rpoS* DMC #1	SRR21604444	RpoS regulon in DMCs	TBDCapSeq	41,404,153	41,366,512	20,838,697	50.38	15,783,241	75.74
1752	Δ*rpoS* DMC #2	SRR21604442		TBDCapSeq	31,318,772	31,291,061	15,091,038	48.23	10,474,216	69.41
1752	Δ*rpoS* DMC #3	SRR21604441		TBDCapSeq	32,529,949	32,506,843	14,754,082	45.39	10,562,186	71.59
1752	Δ*rpoS* DMC #4	SRR21604439		TBDCapSeq	33,197,676	33,168,068	16,012,025	48.28	11,624,596	72.60
1754	*rpoScomp* DMC #1	SRR21604438	RpoS regulon in DMCs	TBDCapSeq	19,335,323	19,320,062	8,623,334	44.63	6,310,391	73.18
1754	*rpoScomp* DMC #2	SRR21604436		TBDCapSeq	36,614,933	36,581,209	17,507,283	47.86	12,837,531	73.33
1754	*rpoScomp* DMC #3	SRR21604435		TBDCapSeq	37,129,967	37,095,669	17,985,661	48.48	13,416,431	74.60
1754	*rpoScomp* DMC #4	SRR21604446		TBDCapSeq	38,825,985	38,791,960	20,097,629	51.81	15,554,324	77.39
1473	WT DMC #1	SRR21604433	Effect of liganded-PlzA on RpoS regulon	TBDCapSeq	5,378,544	5,365,317	1,781,094	33.20	817,446	45.90
1473	WT DMC #2	SRR21604432		TBDCapSeq	4,048,304	4,038,802	1,303,165	32.27	544,766	41.80
1473	WT DMC #3	SRR21604431		TBDCapSeq	13,301,801	13,269,271	3,618,024	27.27	1,860,014	51.41

545	*cDGC* DMC #1	SRR21604430	Effect of liganded-PlzA on RpoS regulon	TBDCapSeq	7,688,078	7,671,298	2,081,557	27.13	592,248	28.45
545	*cDGC* DMC #2	SRR21604429		TBDCapSeq	10,659,417	10,637,866	2,980,701	28.02	929,753	31.19
545	*cDGC* DMC #3	SRR21604428		TBDCapSeq	19,653,627	19,606,765	5,126,589	26.15	2,001,995	39.05
557	*cDGCΔplzA* DMC #1	SRR21604427	Effect of liganded-PlzA on RpoS regulon	TBDCapSeq	5,355,871	5,344,163	1,748,364	32.72	929,776	53.18
557	*cDGCΔplzA* DMC #2	SRR21604426		TBDCapSeq	8,862,079	8,842,341	2,977,895	33.68	1,462,070	49.10
557	*cDGCΔplzA* DMC #3	SRR21604425		TBDCapSeq	12,799,441	12,771,100	3,649,323	28.57	2,002,625	54.88
646	*ΔbosRΔrpoS/irpoS* - IPTG DMC #1	SRR21604443	Effect of BosR on RpoS regulon	Conventional (paired-end)	39,723,626	39,583,526	23,486,330	59.33	12,020,466	51.18
646	*ΔbosRΔrpoS/irpoS* - IPTG DMC #2	SRR21604423		Conventional (paired-end)	51,223,522	51,062,136	28,869,864	56.54	15,300,526	53.00
646	*ΔbosRΔrpoS/irpoS* - IPTG DMC #3	SRR21604422		Conventional (paired-end)	43,507,528	43,327,460	30,870,734	71.25	17,779,115	57.59
646	*ΔbosRΔrpoS/irpoS* + IPTG DMC #1	SRR21604421	Effect of BosR on RpoS regulon	Conventional (paired-end)	47,164,490	46,963,780	29,444,296	62.70	17,833,619	60.57
646	*ΔbosRΔrpoS/irpoS* + IPTG DMC #2	SRR21604420		Conventional (paired-end)	39,885,070	39,758,934	32,906,158	82.76	21,646,813	65.78
646	*ΔbosRΔrpoS/irpoS* +IPTG DMC #3	SRR21604419		Conventional (paired-end)	43,374,484	43,206,594	25,426,134	58.85	15,661,889	61.60

Legend for Supplemental Table 2 (.xlsx). Transcripts per million (TPM) values for TBDCapSeq for WT and Δ*rpoS* within DMCs and fed nymphs.

[A]Locus tags, gene names, and product descriptions are based on *B. burgdorferi* strain B31 RefSeq genome annotations and/or UniProt. Detailed strain descriptions are provided in Supplemental Table 10.

Transcript per million (TPM) values for each biological replicate were calculated as described in Supplemental Methods. Complete description of raw data is provided in Supplemental Table 1.

FedNym, fed nymph; DMC, dialysis membrane chamber.

Legend for Supplemental Table 3 (.xlsx). DESeq2 data for all pairwise comparisons used in these studies.

[A]Locus tags, gene names, and product descriptions are based on *B. burgdorferi* strain B31 RefSeq genome annotations and/or UniProt.

[B]Subcellular localization predictions are based on BUSCA (Bologna Unified Subcellular Component Annotator) (30). Lipoprotein designations are based on Setubal *et al*. (31) and/or SignalP 6.0 (32). Outer membrane protein designations are based on Kenedy *et al*. (33).

[C]Based on previously published RpoS regulon for strain B31 determined by conventional RNAseq (8).

Not DE, not differentially expressed; Cyto, cytoplasm; Lipo, lipoprotein; IM, inner membrane; OM, outer membrane.

Legend for Supplemental Table 4 (.xlsx). Expression profiles of regulatory factors annotated in *Borrelia burgdorferi*.

[A]Locus tags, gene names, and product descriptions are based on *B. burgdorferi* strain B31 RefSeq genome annotations and/or UniProt.

N/A, not applicable when the corresponding gene has been deleted by allelic replacement in one of the strains used the comparison.

Supplemental Table 5. ***B. burgdorferi*** **genes that are significantly upregulated by RpoS in both fed nymphs and mammals.**

Locus tag[A]	Gene[A]	Product[A]	Fold-regulation WT vs Δ*rpoS* in Fed Nymph[B]	RpoS-dependency in Fed Nymphs[C]	Fold-regulation WT vs Δ*rpoS* in DMCs[D]	RpoS-dependency in DMCs[E]	PlzA brake[F]	BosR dependent/ enhanced[H]
BBA05		S1 antigen	6691.85	Y, ↑	10.86	Y	-	enhanced
BBA25	*dbpB*	decorin binding protein B	1054.72	Y	266.64	Y	Y	enhanced
BBA66		outer surface protein (Pfam54_60)	564.28	Y, ↑	14.55	Y	-	enhanced
BBA65		BBA65 lipoprotein (Pfam54_60)	507.11	Y, ↑	4.87	Y	-	Y
BBB19	*ospC*	outer surface protein C	420.87	Y	983.52	Y	Y	enhanced
BBA33		lipoprotein	367.08	Y	9.39	Y	-	enhanced
BBA07	*chpA1*	ChpAI protein	310.52	Y, ↑	21.39	Y	-	enhanced
BBA73		antigen P35 (Pfam54_60)	300.51	Y, ↑	39.00	Y	-	enhanced
BBA0078		lipoprotein (BBA72)	253.81	Y	55.70	Y	Y	enhanced
BBP28	*mlpA*	MlpA lipoprotein	252.89	Y, ↑	5.36	Y	-	enhanced
BBM28	*mlpF*	MlpF lipoprotein	252.36	Y, ↑	9.34	Y	-	enhanced
BB0844		lipoprotein	232.39	Y	182.02	Y	-	enhanced
BBA36		lipoprotein	188.10	Y	100.14	Y	-	enhanced
BBF01		ErpD lipoprotein	160.43	Y	25.51	Y, ↑	-	enhanced
BBJ23		hypothetical protein	131.48	Y	18.18	Y, ↑	-	enhanced
BBJ43		hypothetical protein	122.19	Y	3.14	Y, ↑	-	Y
BBJ24		hypothetical protein	112.17	Y	8.80	Y, ↑	-	enhanced
BBM38	*erpK*	ErpK protein (OspF paralog)	107.64	Y	7.83	Y	-	enhanced
BBH41		inner membrane protein, P13	95.18	Y	64.29	Y, ↑	Y	enhanced
BBA04		S2 antigen	91.12	Y, ↑	3.24	Y	-	Y
BBO39	*erpL*	ErpL lipoprotein (OspF paralog)	81.20	Y, ↑	9.86	Y	-	enhanced
BBA34	*oppA5*	oligopeptide ABC transporter periplasmic oligopeptide-binding protein	78.69	Y	31.43	Y, ↑	-	enhanced
BBJ46		hypothetical protein	76.00	Y	5.13	dual	-	enhanced
BB0040	*cheR-1*	chemotaxis protein methyltransferase CheR-1	73.89	Y, ↑	3.78	Y	-	Y
BBA37		hypothetical protein	73.13	Y	34.98	Y, ↑	Y	enhanced
BBJ29		hypothetical protein	46.44	Y	5.33	Y, ↑	-	Y
BBO40	*erpM*	ErpM lipoprotein (Elp paralog)	45.27	Y, ↑	3.82	Y	-	Y
BBJ26		ABC transporter ATP-binding protein	41.83	Y	9.23	Y, ↑	Y	enhanced
BBA24	*dbpA*	decorin binding protein A	40.30	Y	47.97	Y, ↑	Y	enhanced
BBK53		outer membrane protein	39.66	Y	3.07	dual	-	Y
BBI42		lipoprotein	39.25	Y	5.72	dual	-	enhanced
BBJ25		hypothetical protein	30.19	Y	10.75	Y, ↑	Y	enhanced
BBQ47	*erpX*	ErpX lipoprotein	28.50	Y	17.36	Y, ↑	-	Y
BBM27	*revA*	rev protein	27.92	Y	16.28	Y, ↑	-	enhanced
BBK32		fibronectin-binding protein	23.10	Y	18.64	Y, ↑	Y	enhanced
BBJ28		hypothetical protein	21.70	Y	5.75	Y, ↑	-	enhanced
BBK07		lipoprotein	21.50	Y	7.23	Y	-	enhanced
BB0689		lipoprotein	17.36	Y, ↑	4.02	Y	-	Y
BBJ27		efflux ABC transporter permease	17.29	Y	6.14	dual, ↑	-	enhanced

BB0681	*mcp5*	methyl-accepting chemotaxis protein Mcp5	14.75	dual	6.46	dual	-	enhanced
BB0680	*mcp4*	methyl-accepting chemotaxis protein Mcp4	14.62	dual, ↑	6.16	dual	-	enhanced
BBQ03		lipoprotein	10.51	Y	3.18	dual, ↑	-	Y
BBF0041	*vlsE*	outer surface protein VlsE1	10.36	Y	8.80	Y, ↑	-[G]	Y
BBP27	*revA*	surface protein	10.11	Y	15.54	Y, ↑	-	Y
BB0566		hypothetical protein	8.11	dual	5.21	dual	-	Y
BB0567	*cheA-1*	chemotaxis histidine kinase CheA-1	7.07	dual	5.39	dual	-	Y
BB0798		competence protein F	6.69	Y	3.02	dual	-	Y
BB0565	*cheW-2*	purine-binding chemotaxis protein CheW-2	6.14	dual	5.65	dual	-	Y
BBS42	*bapA*	BapA protein	5.99	Y	3.87	Y, ↑	-	Y
BB0400		hypothetical protein	4.54	Y	4.10	dual	-	Y
BB0671	*cheX*	chemotaxis protein CheX	3.69	dual	3.19	dual	-	Y
BB0563		lipoprotein	3.12	dual	7.06	dual	-	Y

[A]Locus tags, gene names, and product descriptions are based on *B. burgdorferi* strain B31 RefSeq genome annotation and/or UniProt.

[B]Folds of regulation are based on WT vs. Δ*rpoS* Fed Nymph comparison (Supplemental Table 3). Only genes showing ≥ 3-fold higher expression ($q < 0.05$) in WT compared to Δ*rpoS* mutant in fed nymphs are shown.

[C]RpoS dependency is based on previously published studies and/or qualitative assessment of average TPM values for individual genes (Supplemental Table 2). "Y" designates genes that are known or predicted to be transcribed exclusively by RpoS. "Dual" designates genes that appear to be dually-transcribed by RpoD and RpoS. Up arrows (↑) designate genes with enhanced expression (≥ 3-fold; $q < 0.05$) in nymphs compared to DMCs (Supplemental Table 3, WT Fed Nymph vs. DMC comparison).

[D]Folds of regulation are based on WT vs. Δ*rpoS* DMC comparison (Supplemental Table 3). Only genes showing ≥ 3-fold higher expression ($q < 0.05$) in WT compared to Δ*rpoS* mutant are shown.

[E]RpoS dependency is based on previously published studies and/or qualitative assessment of average TPM values for individual genes (Supplemental Table 2). "Y" designates genes that are known or predicted to be transcribed exclusively by RpoS. "Dual" designates genes that appear to be dually-transcribed by RpoD and RpoS. Up arrows (↑) designate genes with enhanced expression (≥ 3-fold; $q < 0.05$) in DMCs compared to nymphs (Supplemental Table 3, WT DMC vs. Fed Nymph comparison).

[F]"Y" designates genes expressed at ≥3-fold ($q < 0.05$) lower levels in the presence of c-di-GMP in DMCs (*cDGC* vs. WT comparison; Supplemental Table 3) but were restored to WT levels in the absence of PlzA (*cDGC*Δ*plz*A vs. WT comparison; Supplemental Table 3). "-" designates genes which expression is not significantly downregulated in the presence of c-di-GMP (*cDGC* vs. WT comparison; Supplemental Table 3).

[G]*vlsE* is downregulated by c-di-GMP (*cDGC* vs. WT comparison; Supplemental Table 3) in a PlzA-independent manner (*cDGC*Δ*plz*A vs. WT comparison; Supplemental Table 3).

[H]"Y" designates RpoS-upregulated genes that require BosR for activation in DMCs (*i.e.*, expressed at comparable levels in Δ*bosR*Δ*rpoS*/*irpoS* +IPTG vs. Δ*bosR*Δ*rpoS/irpoS* –IPTG comparison; Supplemental Table 3). "Enhanced" designates RpoS-upregulated genes whose transcription is enhanced by BosR in DMCs (based on folds of regulation in DMCs for WT vs. Δ*rpoS* and Δ*bosR*Δ*rpoS*/*irpoS* + vs. – IPTG comparisons; Supplemental Table 3)

Supplemental Table 6. ***B. burgdorferi*** **genes that are significantly upregulated by RpoS only within feeding nymphs.**

Locus tag[A]	Gene[A]	Product[A]	Fold-regulation WT vs Δ*rpoS* in Fed Nymph[B]	RpoS-dependency in Fed Nymphs[C]	Fold-regulation WT vs Δ*rpoS* in DMCs[D]	RpoS-dependency in DMCs[E]	PlzA brake[F]
BBE31		P35 antigen (Pfam54_60)	135.55	Y, ↑	1.54	dual	
BBP35	*bppA*	protein BppA	52.96	Y	1.97	dual	-
BBA64		P35 antigen (Pfam54_60)	43.49	Y, ↑	-3.19	RpoD	-
BBR43		hypothetical protein	43.26	Y	-1.19	RpoD, ↑	-
BBQ43	*bppA*	protein BppA	40.34	Y	2.08	dual	-
BBC05		hypothetical protein	37.15	Y	-1.85	RpoD	-
BBK48		immunogenic protein P37	33.50	Y	2.39	dual	-
BBQ37		hypothetical protein	31.08	Y	1.81	dual	-
BBJ47		hypothetical protein	22.22	Y	2.50	dual	-
BBP29		hypothetical protein	20.59	Y	-1.23	RpoD	-
BBJ48		hypothetical protein	20.32	Y	2.96	dual	-
BBJ45		lipoprotein	17.67	Y	1.72	dual, ↑	-
BBJ31		hypothetical protein	17.44	Y	2.15	dual, ↑	-
BBC12		hypothetical protein	16.94	Y	-1.90	RpoD	-
BBK01		lipoprotein	14.74	Y, ↑	-27.10	RpoD	-[G]
BBS41	*erpG*	outer surface protein ErpG (OspF paralog)	13.85	Y	2.83	dual	-
BBQ44	*bppB*	protein BppB	13.33	Y	1.08	RpoD	-
BBA57		P45-13	13.13	Y	1.54	dual	-
BBK0058		hypothetical protein	12.68	Y	-1.09	RpoD	-
BB0418	*dipA*	pore-forming outer membrane protein	11.86	dual, ↑	2.65	dual	-
BBH32		antigen P35	11.26	Y, ↑	-4.40	RpoD	-[G]
BBK17		adenine deaminase	11.24	Y	1.42	dual	-
BBP41		hypothetical protein	10.30	Y	-1.63	RpoD, ↑	-
BBK50		immunogenic protein P37	9.89	Y	-1.37	RpoD	-
BBB09		lipoprotein	9.71	Y	1.04	RpoD	-
BBM39		hypothetical protein	9.14	Y	1.64	dual	-
BBP38	*erpA*	ErpA lipoprotein (OspE paralog)	8.41	Y	-1.86	RpoD	-[G]
BBR44		hypothetical protein	8.24	Y	1.27	RpoD	-
BBR41	*ospE*	outer surface protein E	8.11	Y	1.03	RpoD	-
BB0776		hypothetical protein	7.41	Y, ↑	1.77	dual	-
BB0797	*mutS*	DNA mismatch repair protein MutS	7.36	Y, ↑	1.99	dual	-
BBP39	*erpB*	ErpB lipoprotein	7.20	Y	-1.86	RpoD	-[G]
BBR42	*erpY*	ErpY lipoprotein	7.17	Y	1.16	RpoD, ↑	-
BBR45		phage terminase large subunit	6.28	Y	1.59	dual	-
BBK42		hypothetical protein	6.01	Y	-1.68	RpoD, ↑	-
BB0404		hypothetical protein	5.95	Y	1.04	RpoD	-
BB0777	*apt*	adenine phosphoribosyltransferase	5.92	Y	1.68	dual	-
BBP10		hypothetical protein	5.66	Y	1.85	dual, ↑	-
BBI06		MTA/SAH nucleosidase	5.47	Y	2.26	dual	Y
BBH09		type II restriction enzyme methylase subunit	3.73	dual	2.44	dual, ↑	-
BB0637	*nhaC-1*[H]	Na+/H+ antiporter family	3.73	dual, ↑	1.45	dual	-

BB0729	*gltP*	dicarboxylate/amino acid:cation symporter	3.34	dual, ↑	2.56	dual	-
BBN38	*erpP*	ErpP lipoprotein (OspE paralog)	3.09	Y	-1.69	RpoD	-
BB0045		P115 protein	3.05	Y	1.30	dual	-

[A]Locus tags, gene names, and product descriptions are based on *B. burgdorferi* strain B31 RefSeq genome annotation and/or UniProt.

[B]Folds of regulation are based on WT vs. Δ*rpoS* Fed Nymph comparison (Supplemental Table 3). Only genes showing ≥ 3-fold higher expression ($q < 0.05$) in WT compared to Δ*rpoS* mutant in fed nymphs are shown.

[C]RpoS dependency is based on previously published studies and/or qualitative assessment of average TPM values for individual genes (Supplemental Table 2). "Y" designates genes that are known or predicted to be transcribed exclusively by RpoS. "Dual" designates genes that appear to be dually-transcribed by RpoD and RpoS. Up arrows (↑) designate genes with enhanced expression (≥ 3-fold; $q < 0.05$) in nymphs compared to DMCs (Supplemental Table 3, WT Fed Nymph vs. DMC).

[D]Folds of regulation are based on WT vs. Δ*rpoS* DMC comparison (Supplemental Table 3).

[E]RpoS dependency is based on previously published studies and/or qualitative assessment of average TPM values for individual genes (Supplemental Table 2). "Y" designates genes that are known or predicted to be transcribed exclusively by RpoS. "Dual" designates genes that appear to be dually-transcribed by RpoD and RpoS. RpoD designates genes that appear to be transcribed exclusively by RpoD. Up arrows (↑) designate genes with enhanced expression (≥ 3-fold; $q < 0.05$) in DMCs compared to nymphs (Supplemental Table 3, WT DMC vs. Fed Nymph comparison).

[F]"Y" designates genes expressed at ≥3-fold ($q < 0.05$) lower levels in the presence of c-di-GMP in DMCs (*cDGC* vs. WT comparison; Supplemental Table 3) but were restored to WT levels in the absence of PlzA (*cDGC*Δ*plz*A vs. WT comparison; Supplemental Table 3).

[G]Genes expressed at ≥3-fold ($q < 0.05$) higher levels in the presence of c-di-GMP in DMCs (*cDGC* vs. WT comparison; Supplemental Table 3) but were restored to WT levels in the absence of PlzA (*cDGC*Δ*plz*A vs. WT comparison; Supplemental Table 3).

[H]*bb0638*/*nhaC-2* also was upregulated 2.98-fold ($q < 0.05$) by RpoS in fed nymphs (Supplemental Table 3) but missed the 3-fold cut off required for inclusion in the RpoS regulon. Neither *nhaC-1* nor *nhaC-2* was upregulated by RpoS in DMCs (Supplemental Table 3).

Supplemental Table 7. ***B. burgdorferi*** **genes significantly upregulated by RpoS only in DMCs.**

Locus tag[A]	Gene[A]	Product[A]	Fold-regulation WT vs Δ*rpoS* in DMCs[B]	RpoS-dependency in DMCs[C]	Fold-regulation WT vs Δ*rpoS* in Fed Nymphs[D]	RpoS-dependency in Fed Nymphs[E]	PlzA brake[F]	BosR dependent/ enhanced[G]
BBG27		hypothetical protein	201.87	Y, ↑	11.27*	<10 TPM	Y	enhanced
BBG28		hypothetical protein	137.05	Y, ↑	2.21	<10 TPM	Y	Y
BBG25		lipoprotein	127.20	Y, ↑	41.69*	<10 TPM	Y	enhanced
BBG26		hypothetical protein	112.34	Y, ↑	6.68*	<10 TPM	-	enhanced
BBG24		hypothetical protein	43.05	Y, ↑	7.14*	<10 TPM	-	enhanced
BBG22		hypothetical protein	39.55	Y, ↑	6.57*	<10 TPM	-	enhanced
BBG15		hypothetical protein	25.15	Y, ↑	7.40*	<10 TPM	-	Y
BBG16		hypothetical protein	21.13	Y, ↑	40.91*	Y	-	Y
BBG14		hypothetical protein	20.12	Y, ↑	3.27*	<10 TPM	-	Y
BBG18		hypothetical protein	19.14	Y, ↑	1.90	dual	-	Y
BBG19		hypothetical protein	17.52	dual, ↑	3.77*	Y	-	Y
BBG23		hypothetical protein	17.46	Y, ↑	17.31*	<10 TPM	-	Y
BBG17		hypothetical protein	17.45	Y, ↑	4.90*	<10 TPM	-	Y
BBG20		hypothetical protein	16.10	Y, ↑	5.62*	<10 TPM	-	Y
BBG13		hypothetical protein	12.49	dual, ↑	4.48*	<10 TPM	-	Y
BBG21		hypothetical protein	11.44	Y, ↑	1.58	<10 TPM	-	Y
BBG12		hypothetical protein	9.92	dual, ↑	14.59*	<10 TPM	-	Y
BBG0036		hypothetical protein	8.88	Y, ↑	9.93*	<10 TPM	-	Y
BBG31		hypothetical protein	7.95	dual, ↑	7.38*	Y	-	Y
BBG32		replicative DNA helicase	7.29	Y, ↑	1.57	<10 TPM	-	Y
BBD24		hypothetical protein	6.20	Y, ↑	-1.00	<10 TPM	Y	-
BBG29		hypothetical protein	6.05	dual, ↑	1.95	<10 TPM	-	Y
BB0116	*malX-1*	PTS system maltose and glucose-specific transporter subunit IIABC	5.90	Y, ↑	1.16	RpoD	-	Y
BBG30		hypothetical protein	5.05	Y, ↑	1.09	<10 TPM	-	Y
BBH40		transposase-like protein	4.05	Y, ↑	22.60*	<10 TPM	-	Y
BBD0031		hypothetical protein	3.94	Y, ↑	3.47*	<10 TPM	-	-
BBT07		hypothetical protein	3.86	Y, ↑	-1.00	<10 TPM	-	Y
BB0287	*flbA*	flagellar protein FlbA	3.81	Y, ↑	1.02	RpoD	Y	Y
BBA32		lipoprotein	3.76	Y	5.62*	Y	-	Y
BB0548	*polA*	DNA polymerase I	3.75	dual	1.27	RpoD	-	Y
BB0208		hypothetical protein	3.59	Y	1.90	<10 TPM	-	Y
BB0580		integral membrane protein	3.43	dual, ↑	-1.52	RpoD	Y	Y
BB0547	*coaE*	dephospho-CoA kinase	3.36	Y, ↑	-1.43	RpoD	-	Y

BB0669	*cheA-2*	chemotaxis protein CheA-2	3.20	dual	1.51	RpoD	-	Y
BB0670	*cheW-3*	purine-binding chemotaxis protein CheW-3	3.20	dual	2.51	RpoD	-	Y
BB0273	*fliR*	flagellar biosynthetic protein FliR	3.18	dual, ↑	-1.02	RpoD	-	Y
BBK33		hypothetical protein	3.17	Y, ↑	10.86*	Y	-	Y
BB0274	*fliQ*	flagellar biosynthesis protein FliQ	3.11	dual, ↑	1.13	RpoD	-	Y
BB0578	*mcp-1*	methyl-accepting chemotaxis protein Mcp1	3.06	dual, ↑	-1.21	RpoD	Y	Y
BB0581	*recG*	ATP-dependent DNA helicase RecG	3.00	dual, ↑	-1.26	RpoD	-	Y

[A]Locus tags, gene names, and product descriptions are based on *B. burgdorferi* strain B31 RefSeq genome annotation and/or UniProt.

[B]Folds of regulation are based on WT vs. Δ*rpoS* DMC comparison (Supplemental Table 3). Only genes showing ≥ 3-fold higher expression ($q < 0.05$) in WT compared to Δ*rpoS* mutant are shown.

[C]RpoS dependency is based on previously published studies and/or qualitative assessment of average TPM values for individual genes (Supplemental Table 2). "Y" designates genes that are known or predicted to be transcribed exclusively by RpoS. "Dual" designates genes that appear to be dually-transcribed by RpoD and RpoS. Up arrows (↑) designate genes with enhanced expression (≥ 3-fold; $q < 0.05$) in DMCs compared to nymphs (Supplemental Table 3, WT DMC vs. Fed Nymph comparison).

[D]Folds of regulation are based on WT vs. Δ*rpoS* Fed Nymph comparison (Supplemental Table 3). Asterisks (*) designate genes showing ≥ 3-fold higher expression in WT compared to Δ*rpoS* mutant in fed nymphs but not statistically significant ($q > 0.05$).

[E]RpoS dependency is based on previously published studies and/or qualitative assessment of average TPM values for individual genes (Supplemental Table 2). "Y" designates genes that are known or predicted to be transcribed exclusively by RpoS. "Dual" designates genes that appear to be dually-transcribed by RpoD and RpoS. "RpoD" designates genes that appear to be transcribed exclusively by RpoD. Up arrows (↑) designate genes with enhanced expression (≥ 3-fold; $q < 0.05$) in nymphs compared to DMCs (Supplemental Table 3, WT Fed Nymph vs. DMC). <10 TPM designates genes expressed at very low levels (average TPM <10; Supplemental Table 2) by WT *Bb*.

[F]"Y" designates genes expressed at ≥3-fold ($q < 0.05$) lower levels in the presence of c-di-GMP in DMCs (*cDGC* vs. WT comparison; Supplemental Table 3) but were restored to WT levels in the absence of PlzA (*cDGC*Δ*plz*A vs. WT comparison; Supplemental Table 3). "-" designates genes which expression is not significantly downregulated in the presence of c-di-GMP (*cDGC* vs. WT comparison; Supplemental Table 3).

[G]"Y" designates RpoS-upregulated genes that require BosR for activation in DMCs (*i.e.*, expressed at comparable levels in Δ*bosR*Δ*rpoS*/*irpoS* +IPTG vs. Δ*bosR*Δ*rpoS/irpoS* –IPTG comparison; Supplemental Table 3). "Enhanced" designates RpoS-upregulated genes whose transcription is enhanced by BosR in DMCs (based on folds of regulation for WT vs. Δ*rpoS*-DMC and Δ*bosR*Δ*rpoS*/*irpoS* + vs. –IPTG comparisons; Supplemental Table 3). "-" designates genes upregulated by RpoS independently of BosR (*i.e.,* genes expressed at ≥3-fold [$q < 0.05$] higher levels in Δ*bosR*Δ*rpoS*/*irpoS* +IPTG vs. Δ*bosR*Δ*rpoS/irpoS* –IPTG comparison; Supplemental Table 3).

Supplemental Table 8. ***B. burgdorferi*** **genes repressed by RpoS in mammals.**

Locus Tag[A]	Gene[A]	Product[A]	Fold-regulation WT vs Δ*rpoS* in DMCs[B]	Tick phase gene[C]	PlzA brake[D]	BosR dependency[E]
BBJ09	*ospD*	outer surface protein D	-89.29	Y	Y	Y
BBJ08		surface protein	-61.81	Y	Y	Y
BBA68	*BbCRASP-1*	complement regulator-acquiring surface protein 1 (Pfam54_60)	-58.41	Y	Y	Y
BBH37		lipoprotein	-55.17	Y	Y	Y
BBJ41		antigen P35 (Pfam54_60)	-49.69	Y	Y	Y
BBA15	*ospA*	outer surface protein A	-45.99	Y	Y	Y
BBA62	*lp6.6*	6.6 kDa lipoprotein	-37.97	Y	Y	Y
BBA16	*ospB*	outer surface protein B	-37.87	Y	Y	Y
BBA74	*bba74*	osm28	-37.86	Y	Y	Y
BBA69		putative surface protein (Pfam54_60)	-34.08	Y	Y	Y
BBA38		phage portal protein	-26.02	Y	Y	Y
BBA61		hypothetical protein	-17.92	Y	Y	Y
BBA40		hypothetical protein	-15.31	Y	Y	Y
BB0242	*orf*	hypothetical protein	-11.16	Y	Y	-
BB0631		hypothetical protein	-10.44	Y	Y	Y
BB0240	*glpF*	glycerol uptake facilitator GlpF	-9.76	Y	Y	Y
BBA59		lipoprotein	-8.64	Y	Y	Y
BB0241	*glpK*	glycerol kinase GlpK	-7.24	Y	Y	Y
BBA03		lipoprotein	-5.88	Y	Y	Y
BB0243	*glpA*	glycerol-3-phosphate dehydrogenase GlpA	-5.38	Y	Y	Y
BB0034	*p13*	outer membrane protein P13	-5.20	Y	Y	Y
BB0365	*la7*	lipoprotein LA7	-5.10	Y	Y	Y
BB0330	*oppA3*	oligopeptide ABC transporter periplasmic oligopeptide-binding protein (OppA-3)	-4.57	Y	Y	Y
BBB29	*malX-2*	PTS system transporter subunit IIBC	-3.56	Y	Y	-
BBA60		surface lipoprotein P27	-3.10	Y	Y	Y
BBA52		outer membrane protein	-3.06	Y	Y	Y
BB0084	*nifS*	cysteine desulfurase	-4.93	Y	-	Y

BBI29		virulence associated lipoprotein	-3.81	Y	-	Y
BBI16	*vraA*	virulence associated lipoprotein VraA	-3.22	Y	-	Y
BB0028		lipoprotein	-3.15	Y	-	Y
BBI39		surface antigen (Pfam54_60)	-47.74	-	Y	Y
BBD18		hypothetical protein	-41.50	-	Y	Y
BBK15		antigen P35	-31.34	-	Y	Y
BBG01		lipoprotein	-18.64	-	Y	Y
BBI38		surface antigen (Pfam54_60)	-14.50	-	Y	Y
BBA41		hypothetical protein	-12.73	-	Y	Y
BBI36		antigen P35 (Pfam54_60)	-10.87	-	Y	Y
BBA42		hypothetical protein	-9.52	-	Y	Y
BBK45		immunogenic protein P37	-6.90	-	Y	Y
BBA43		hypothetical protein	-5.54	-	Y	Y
BBR27	*bdrH*	BdrH	-5.14	-	Y	Y
BBK13		hypothetical protein	-5.10	-	Y	Y
BBH26		hypothetical protein	-4.65	-	Y	Y
BBA54		hypothetical protein	-4.50	-	Y	Y
BBA53		Bbs27 protein	-4.47	-	Y	Y
BBA14		lipoprotein	-4.35	-	Y	Y
BBA45		hypothetical protein	-4.32	-	Y	Y
BBK23		hypothetical protein	-4.26	-	Y	Y
BBF17		putative transmembrane protein	-4.06	-	Y	Y
BBH13		protein RepU	-3.95	-	Y	Y
BBK22		hypothetical protein	-3.93	-	Y	Y
BBL27	*bdrP*	protein BdrP	-3.93	-	Y	Y
BBK40		hypothetical protein	-3.74	-	Y	Y
BBA46		hypothetical protein	-3.60	-	Y	Y
BBH27		hypothetical protein	-3.52	-	Y	Y
BBG02		hypothetical protein	-3.38	-	Y	Y
BBR28	*mlpD*	lipoprotein	-3.36	-	Y	Y
BBJ19		hypothetical protein	-3.29	-	Y	Y
BBH0042		hypothetical protein	-3.22	-	Y	Y

BBA47		hypothetical protein	-3.12	-	Y	Y
BBH25		hypothetical protein	-3.12	-	Y	Y
BBC11		hypothetical protein	-6.02	-	-	Y
BBU02		hypothetical protein	-4.69	-	-	Y
BBR03		hypothetical protein	-4.57	-	-	Y
BB0159		hypothetical protein	-3.67	-	-	Y
BBR04		hypothetical protein	-3.60	-	-	Y
BBJ11		hypothetical protein	-3.53	-	-	Y
BBC10	*revB*	rev protein	-3.53	-	-	Y
BBK24	*pf49*	PF-49 protein	-3.39	-	-	Y
BBK41		hypothetical protein	-3.31	-	-	Y
BB0454		lipopolysaccharide biosynthesis-like protein	-3.29	-	-	Y
BBF20		lipoprotein	-3.28	-	-	Y
BBC02		hypothetical protein	-3.28	-	-	Y
BBK35		hypothetical protein	-3.24	-	-	Y
BBC04		hypothetical protein	-3.16	-	-	Y
BBR05		hypothetical protein	-3.12	-	-	Y
BBF06		hypothetical protein	-3.06	-	-	Y

[A]Locus tags, gene names, and product descriptions are based on *B. burgdorferi* strain B31 RefSeq genome annotation and/or UniProt.

[B]Folds of regulation are based on WT vs. Δ*rpoS* DMC comparison (Supplemental Table 3).

[C]"Y" designates known or putative tick phase genes (*i.e.*, expressed by WT- and Δ*rpoS*-infected fed nymphs and strongly repressed by RpoS in DMCs). "-" designates genes that are not tick-phase genes.

[D]"Y" designates genes expressed at ≥3-fold ($q < 0.05$) higher levels in the presence of c-di-GMP (*cDGC* vs. WT comparison; Supplemental Table 3) but were restored to WT levels in the absence of PlzA (*cDGC*Δ*plzA* vs. WT comparison; Supplemental Table 3). "-" designates genes which expression is not significantly upregulated in the presence of c-di-GMP (*cDGC* vs. WT comparison; Supplemental Table 3).

[E]"Y" designates RpoS-repressed genes that require BosR based on Δ*bosR*Δ*rpoS*/*irpoS* +IPTG vs. –IPTG comparison (Supplemental Table 3). "-" designates genes repressed by RpoS independently of BosR (*i.e.,* genes expressed at ≥3-fold [$q < 0.05$] lower levels in Δ*bosR*Δ*rpoS*/*irpoS* +IPTG vs. Δ*bosR*Δ*rpoS/irpoS* –IPTG comparison; Supplemental Table 3)

Legend for Supplemental Table 9 (.xlsx). RpoS-independent genes differentially expressed by *Bb* in fed nymphs and DMCs defined by TBDCapSeq.

[A]Locus tags, gene names, and product descriptions are based on *B. burgdorferi* strain B31 RefSeq genome annotations and/or UniProt.

[B]Genes expressed at ≥3-fold ($q < 0.05$) higher levels by WT *Bb* in DMCs compared to fed nymphs (Supplemental Table 3, WT DMC vs. Fed Nymph comparison).

[C]Genes expressed at ≥3-fold ($q < 0.05$) higher levels by WT *Bb* in fed nymphs compared to DMCs (Supplemental Table 3, WT Fed Nymph vs. DMC comparison).

Supplemental Table 10. Bacterial strains used in these studies.

Strain number	Strain name	Description	Antibiotic Resistance[A]	Reference
BbP1781	WT	B31 5A4 wild-type parent	none	(8)
BbP1752	Δ*rpoS*	B31 5A4 (BbP1781) containing an insertion in *rpoS*	Streptomycin	(8)
BbP1754	*rpoS*comp	Δ*rpoS* (BbP1752) *trans*-complemented with a wild-type copy of *rpoS* under the native promoter	Streptomycin Kanamycin	(8)
BbAG351	Δ*rpoS*/ *irpoS*	Δ*rpoS* (BbP1752) complemented with an IPTG-inducible *rpoS* allele (*irpoS*) inserted into the endogenous cp26 plasmid	Streptomycin Gentamicin	This study
BbAG646	Δ*bosR* Δ*rpoS*/ *irpoS*	BbAG351 containing an insertion in *bosR*	Streptomycin Kanamycin Gentamicin	This study
OY10	Δ*bosR*	B31 MI Δ*bosR*	Kanamycin	(9)
BbAG580	Δ*bosR*/ *irpoS*	B31 MI Δ*bosR* containing an IPTG-inducible *rpoS* allele (*irpoS*) inserted into the endogenous cp26 plasmid	Kanamycin Gentamicin	This study
BbAG643	*bosRcomp*/ *irpoS*	Δ*bosR*+*irpoS* (BbAG580) strain *cis*-complemented for *bosR*	Streptomycin Gentamicin	This study
BbP1473	WT B31 A3-68	B31 A3 containing an insertion in *bbe02;* reisolated from an infected mouse; wild-type parent for *cDGC* and *cDGC*Δ*plzA* strains	Streptomycin	(39, 40)
BbAG545	*cDGC*	B31 A3-68 encoding a constitutively active diguanylate cyclase (P*flaB*-*slr1143-HA*) inserted into the native *rrp1* locus by allelic replacement	Streptomycin Gentamicin	This study
BbAG557	*cDGC* Δ*plzA*	B31 A3-68 Δ*plzA* (BbP1474) encoding a constitutively active diguanylate cyclase (P*flaB*-*slr1143-HA*) inserted into the native *rrp1* locus by allelic replacement	Streptomycin Kanamycin Gentamicin	(2)

[A]Antibiotic resistance refers to selection in *B. burgdorferi*. P*flgB*::*aadA* cassette confers resistance to streptomycin and spectinomycin in *B. burgdorferi* and *E. coli.*

Supplemental Table 11. Oligonucleotide primers used in these studies.

Primer	5' – 3' sequence	Purpose	Reference
rpoS-5'	GGAGAAATTACATATGAACATATTTAGTAATGAGGATTTAA ACATATATT	Construction of inducible *rpoS* allele (*irpoS*)	This study
rpoS-3'	CTCTATCTTCAAGCTTTTAATTTATTTCTTCTTTTAATTTTTT AAGAACTCTT	Construction of inducible *rpoS* allele (*irpoS*)	This study
irpoS-*lacI*-5'	TCGGGTAGGATCCCGACGTCTCTAGAAAATCATAAAAAATT TATTTGCTT	Insertion of *irpoS*-*lacI* cassette into cp26	This study
irpoS-*lacI*-3'	CAAAATTTCTAGATGACGTCTTATTACTGGCCGCTTTCTAG	Insertion of *irpoS*-*lacI* cassette into cp26	This study
bosR-5'	CGACTCTAGAGGATCCGATCCAAACTTACCACCGAACTACT AGAG	Cloning *bosR* plus flanking regions	This study
bosR-3'	CGGTACCCGGGGATCCGGCAATGGGGTTCAGGTAGTTTACG GACCAGGTG	Cloning *bosR* plus flanking regions	This study
invpUC *bosR*-5'	ATGAATATAAAAAATATCATTTTTATACTTATATTC	Linearization of pUC/*bosR*	This study
invpUC *bosR*-3'	ATGATTATACCTTTTTTGTTTAAATTAAAG	Linearization of pUC/*bosR*	This study
bosR-*kanR*-5'	AAAAGGTATAATCATTACCCGAGCTTCAAGGAAGA	Replace bosR with *PflgB-kanR*	This study
bosR-*kanR*-3'	ATTTTTTATATTCATTTAGAAAAACTCATCGAGCATCA	Replace *bos*R with *PflgB-kanR*	This study
*bosR*comp SS-5'	GAAATCACTTTATGAAGATCTCAGCTTTTTTTTGAAGTGCCT	Construction of *bosR* complement	This study
*bosR*comp SS-3'	ATTTTTTATATTCATTTTGCCGACTACCTTGGTGATCTC	Construction of *bosR* complement	This study
invpUC *bosR* comp-3'	AAAAAGCTGAGATCTTCATAAAGTGATTTCCTTGTTCTCAT CTGGG	Linearization of pUC/*bosR*	This study
bbd18-260-468-5'	TGCAAACCGGTGAAAATTACG	qRT-PCR	This study
bbd18-260-468-3'	AATTTCTTCTGCAGTTGGTTCAT	qRT-PCR	This study
flaB-F	CTTTTCTCTGGTGAGGGAGCTC	qRT-PCR	(41)
flaB-R	GCTCCTTCCTGTTGAACACCC	qRT-PCR	(41)
flaB-probe	[6FAM]CTTGAACCGGTGCAGCCTGAGCA[BHQ1]	qRT-PCR	(41)

Supplemental Table 12. Bacterial plasmids used in these studies.

Plasmid name	Description	Antibiotic resistance[A]	Ref
pJSB275	cp9-based *E. coli-B. burgdorferi* shuttle vector encoding luciferase (*luc*) under the control of the IPTG-inducible T5 promoter from pQE30	Streptomycin	(6)
pJSB275/ *irpoS*	pJSB275 encoding an inducible *rpoS* allele generated by replacing the *luc* gene in pJSB275	Streptomycin	This study
EcAG265	pUC19-based empty starting vector encoding a P*flgB-aacA* cassette; used to insert sequences of interest into the endogenous cp26 plasmid of *B. burgdorferi* strain B31	Gentamicin	(2)
EcAG291	EcAG265 with *irpoS-lacI* cassette from pJSB275/*irpoS*	Gentamicin	This study
pUC19/ *bosR*	pUC19 containing *bosR* with ~1-kb of up and downstream flanking sequence; used to generate pMC5115	Ampicillin	This study
pBSV2	cp9-based *E. coli-B. burgdorferi* shuttle vector	Kanamycin	(10)
pMC5115	pUC19/*bosR* with P*flgB-kanR* cassette replacing *bosR* coding sequence; used to inactivate *bosR*	Ampicillin Kanamycin	This study
pMC4925	pUC19/*bosR* with P*flgB-aadA* cassette from pJSB275 cloned downstream of *bosR;* used for *cis*-complementation of *bosR*	Ampicillin Streptomycin	This study
EcAG391	pUC19 clone containing P*flaB-slr1143-HA* flanked by ~1-kb of upstream and downstream sequence for *rrp1*; used to generate *cDGC* strains	Ampicillin Gentamicin	(2)

[A]Antibiotic resistance refers to selection in *B. burgdorferi* and/or *E. coli.* P*flgB*::*aadA* cassette confers resistance to streptomycin and spectinomycin in *B. burgdorferi* and *E. coli* (42). Ampicillin resistance gene (*bla*) is used for selection in *E. coli*.

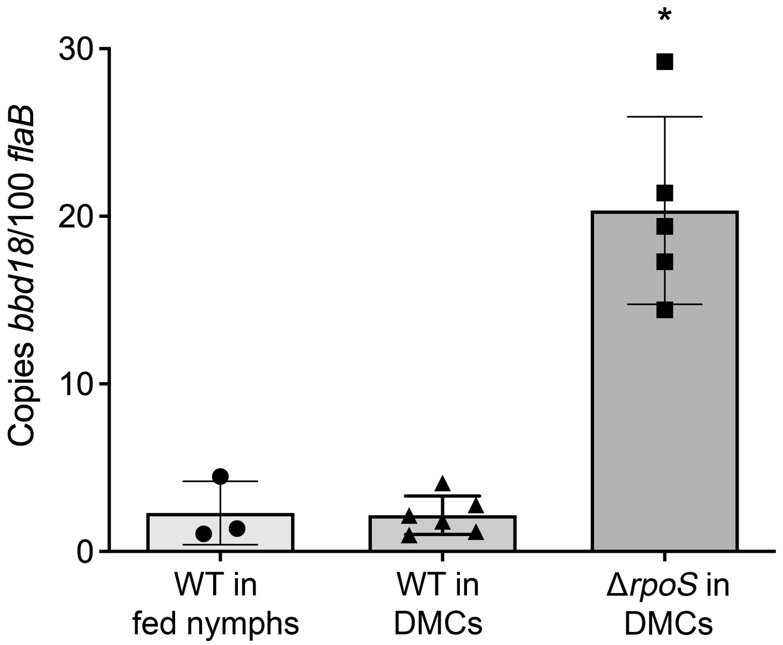

Supplemental Figure 1. Transcription of *bbd18* by RpoD is maintained at low levels in mammals by RpoS-mediated repression. Transcripts for *bbd18* were measured by qRT-PCR for wild-type (WT) *Bb* in engorged nymphs (3 pools, 6-8 nymphs per pool) and WT and Δ*rpoS* Bb cultivated in DMCs (6 and 5 biological replicates, respectively). Transcript copy numbers for *bbd18* were normalized using *bb0147*/*flaB*. Statistical significance was determined by unpaired Student's *t*-test. *, $p<0.05$.

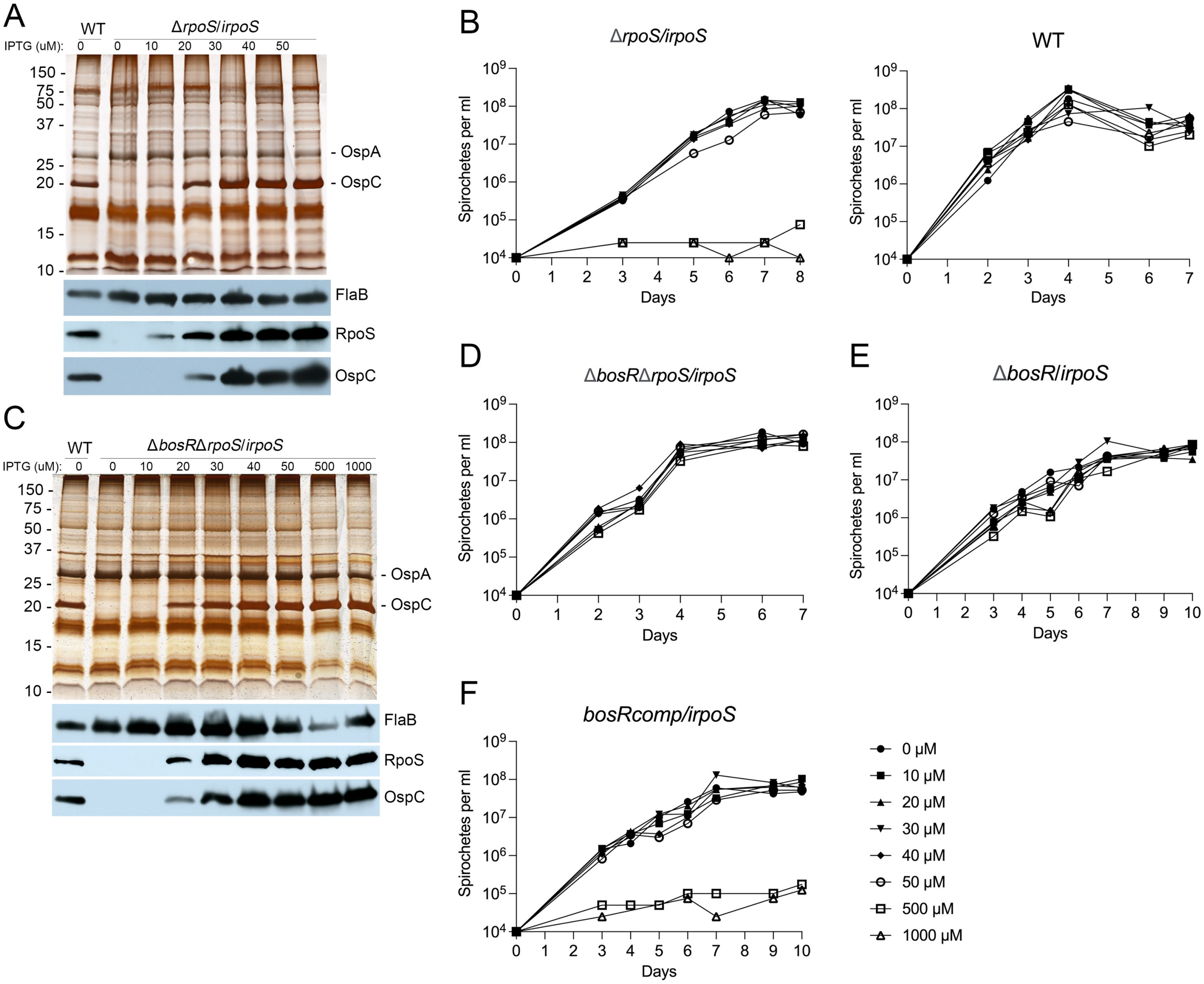

Supplemental Figure 2. IPTG-induction of RpoS circumvents the need for BosR in vitro. **A**. Whole-cell lysates from isogenic wild-type (WT) and Δ*rpoS/irpoS* strains cultivated in vitro with 0 - 50 µM IPTG were separated by SDS-PAGE and stained with silver or immunoblotted with antisera against FlaB, RpoS and OspC. **B**. Growth curves of WT and Δ*rpoS/irpoS* at 37˚C. BSK-II supplemented with increasing concentrations of IPTG. **C**. Whole-cell lysates from WT and Δ*bosR*Δ*rpoS/irpoS* strains cultivated in vitro with 0 - 1000 µM IPTG were separated by SDS-PAGE and stained with silver or immunoblotted with antisera against FlaB, RpoS and OspC. The toxicity observed following over-induction of RpoS is ameliorated in the absence of *bosR*. BSK-II supplemented with increasing concentrations of IPTG were inoculated with Δ*bosR*Δ*rpoS/irpoS* (**D**)*,* Δ*bosR/irpoS* (**E**) and *bosRcomp/irpoS* (**F**) at a starting density of 1 × 10^4 *Bb*/ml. Cultures were maintained at 37°C and enumerated daily until stationary phase (7-10 days). **A-F** show representative images from 3 biological replicates per strain.

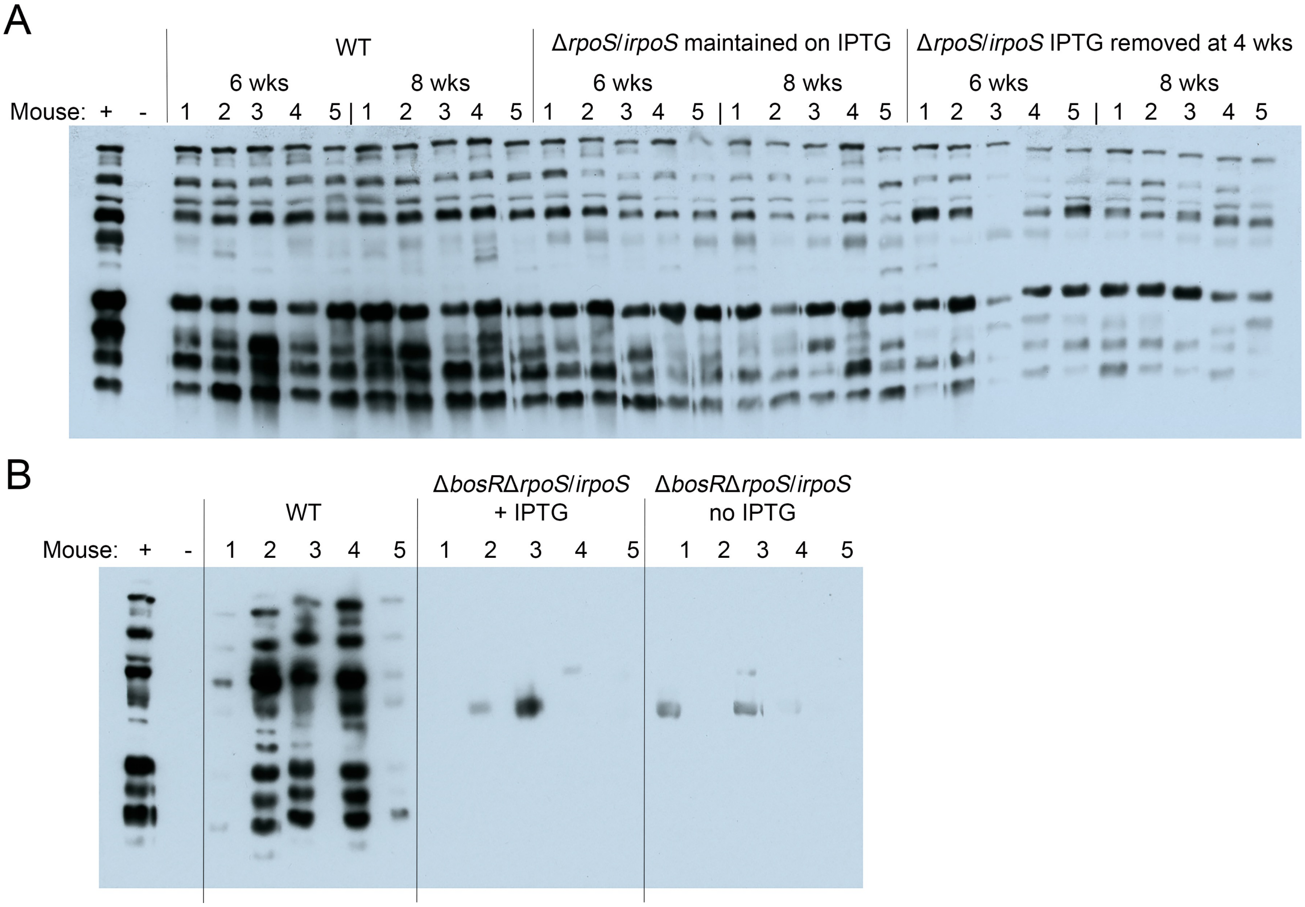

Supplemental Figure 3. Immunoblot analysis of sera collected from C3H/HeJ mice inoculated with wild-type, Δ*rpoS/irpoS* and Δ*bosR*Δ*rpoS/irpoS* four weeks after inoculation. **A**. Sera collected from mice inoculated with isogenic wild-type (WT) and Δ*rpoS/irpoS* strains. As presented in Figure 5A, mice inoculated with Δ*rpoS/irpoS* received IPTG-treated water for the first 4 weeks, then treatment was removed from half of the mice while the remaining half were maintained on IPTG-treated water. **B**. Sera collected from mice inoculated with WT and Δ*bosR*Δ*rpoS/irpoS.* Mice infected with WT *Bb* were maintained on untreated water throughout the entire experiment. One group of mice inoculated with Δ*bosR*Δ*rpoS/irpoS* (+ IPTG) received IPTG-treated water one week prior to infection and then remained on treated water throughout the entire experiment. A second group of Δ*bosR*Δ*rpoS/irpoS* (no IPTG) received only untreated water. In **A** and **B**, sera were diluted 1:1,000 and immunoblotted against *Bb* strain B31 whole cell lysates.

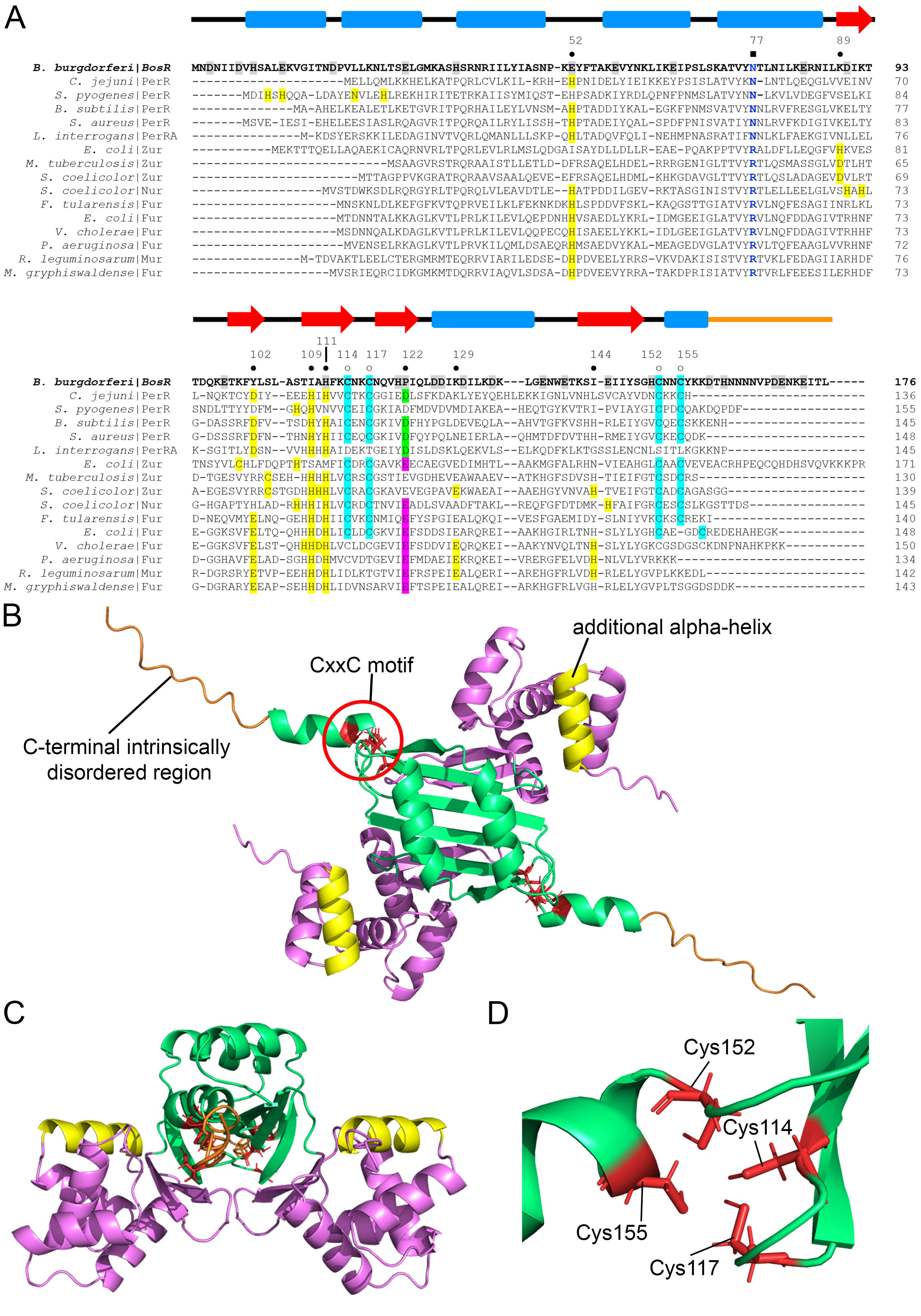
A
52
77
89
B. burgdorferi|BosR MNDNIIDVHSALEKVGITNDPVLLKNLTSELGMKASHSRNRIILYIASNP-KEYFTAKEVYNKLIKEIPSLSKATVYNTLNILKERNILKDIKT 93
C. jejuni|PerR ---------------------MELLQMLKKHELKATPQRLCVLKIL-KRH-EHPNIDELYIEIKKEYPSISLATVYKN-LNTLQEQGLVVEINV 70
S. pyogenes|PerR -------MDIHSHQQA-LDAYENVLEHLREKHIRITETRKAIISYMIQST-EHPSADKIYRDLQPNFPNMSLATVYNN-LKVLVDEGFVSELKI 84
B. subtilis|PerR --------------MA-AHELKEALETLKETGVRITPQRHAILEYLVNSM-AHPTADDIYKAL-EGKFPNMSVATVYNNLRVFRESGLVKELTY 77
S. aureus|PerR -------MSVE-IESI-EHELEESIASLRQAGVRITPQRQAILRYLISSH-THPTADEIYQAL-SPDFPNISVATIYNNLRVFKDIGIVKELTY 83
L. interrogans|PerRA ---------------M-KDSYERSKKILEDAGINVTVQRLQMANLLLSKP-QHLTADQVFQLI-NEHMPNASRATIFNNLKLFAEKGIVNLLEL 76
E. coli|Zur -----------MEKTTTQELLAQAEKICAQRNVRLTPQRLEVLRLMSLQDGAISAYDLLDLLR-EAE-PQAKPPTVYRALDFLLEQGFVHKVES 81
M. tuberculosis|Zur ---------------------------MSAAGVRSTRQRAAISTLLETLD-DFRSAQELHDEL-RRRGENIGLTTVYRTLQSMASSGLVDTLHT 65
S. coelicolor|Zur ------------------------MTTAGPPVKGRATRQRAAVSALQEVE-EFRSAQELHDML-KHKGDAVGLTTVYRTLQSLADAGEVDVLRT 69
S. coelicolor|Nur ------------------MVSTDWKSDLRQRGYRLTPQRQLVLEAVDTLE--HATPDDILGEV-RKTASGINISTVYRTLELLEELGLVSHAHL 73
F. tularensis|Fur --------------------MNSKNLDLKEFGFKVTQPRVEILKLFEKNKDKHLSPDDVFSKL-KAQGSTTGIATVYRVLNQFESAGIINRLKL 73
E. coli|Fur --------------------MTDNNTALKKAGLKVTLPRLKILEVLQEPDNHHVSAEDLYKRL-IDMGEEIGLATVYRVLNQFDDAGIVTRHNF 73
V. cholerae|Fur --------------------MSDNNQALKDAGLKVTLPRLKILEVLQQPECQHISAEELYKKL-IDLGEEIGLATVYRVLNQFDDAGIVTRHHF 73
P. aeruginosa|Fur ---------------------MVENSELRKAGLKVTLPRVKILQMLDSAEQRHMSAEDVYKAL-MEAGEDVGLATVYRVLTQFEAAGLVVRHNF 72
R. leguminosarum|Mur ---------------M-TDVAKTLEELCTERGMRMTEQRRVIARILEDSE-DHPDVEELYRRS-VKVDAKISISTVYRTVKLFEDAGIIARHDF 76
M. gryphiswaldense|Fur -------------------MVSRIEQRCIDKGMKMTDQRRVIAQVLSDSA-DHPDVEEVYRRA-TAKDPRISIATVYRTVRLFEEESILERHDF 73
111
102 109 114 117 122 129 144 152 155
B. burgdorferi|BosR TDQKETKFYLSL-ASTIAHFKCNKCNQVHPIQLDDIKDILKDK---LGENWETKSI-EIIYSGHCNNCYKKDTHNNNNVPDENKEITL----- 176
C. jejuni|PerR L-NQKTCYDIY--EEEHIHVVCTKCGGIEDLSFKDAKLYEYQEHLEKKIGNLVNHLSVCAYVDNCKKCH------------------------ 136
S. pyogenes|PerR SNDLTTYYDFM--GHQHVNVVCEICGKIADFMDVDVMDIAKEA--HEQTGYKVTRI-PVIAYGICPDCQAKDQPDF----------------- 155
B. subtilis|PerR G-DASSRFDFV--TSDHYHAICENCGKIVDFHYPGLDEVEQLA--AHVTGFKVSHH-RLEIYGVCQECSKKENH------------------- 145
S. aureus|PerR G-DSSSRFDFN--THNHYHIICEQCGKIVDFQYPQLNEIERLA--QHMTDFDVTHH-RMEIYGVCKECQDK---------------------- 148
L. interrogans|PerRA K-SGITLYDSN--VVHHHHAIDEKTGEIYDISLDSKLQEKVLS--ELKQDFKLKTGSSLENCNLSITLKGKKNP------------------- 145
E. coli|Zur TNSYVLCHLFDQPTHTSAMFICDRCGAVKEECAEGVEDIMHTL--AAKMGFALRHN-VIEAHGLCAACVEVEACRHPEQCQHDHSVQVKKKPR 171
M. tuberculosis|Zur D-TGESVYRRCSEH-HHHHLVCRSCGSTIEVGDHEVEAWAAEV--ATKHGFSDVSH-TIEIFGTCSDCRS----------------------- 130
S. coelicolor|Zur A-EGESVYRRCSTGDHHHHLVCRACGKAVEVEGPAVEKWAEAI--AAEHGYVNVAH-TVEIFGTCADCAGASGG------------------- 139
S. coelicolor|Nur G-HGAPTYHLAD-RHHHIHLVCRDCTNVIEADLSVAADFTAKL--REQFGFDTDMK-HFAIFGRCESCSLKGSTTDS---------------- 145
F. tularensis|Fur D-NEQVMYELNQ-GEHHDHIICVKCNMIQEFYSPGIEALQKQI--VESFGAEMIDY-SLNIYVKCKSCREKI--------------------- 140
E. coli|Fur E-GGKSVFELTQ-QHHHDHLICLDCGKVIEFSDDSIEARQREI--AAKHGIRLTNH-SLYLYGHCAE-GDCREDEHAHEGK------------ 148
V. cholerae|Fur E-GGKSVFELST-QHHHDHLVCLDCGEVIEFSDDVIEQRQKEI--AAKYNVQLTNH-SLYLYGKCGSDGSCKDNPNAHKPKK----------- 150
P. aeruginosa|Fur D-GGHAVFELAD-SGHHDHMVCVDTGEVIEFMDAEIEKRQKEI--VRERGFELVDH-NLVLYVRKKK-------------------------- 134
R. leguminosarum|Mur R-DGRSRYETVP-EEHHDHLIDLKTGTVIEFRSPEIEALQERI--AREHGFRLVDH-RLELYGVPLKKEDL---------------------- 142
M. gryphiswaldense|Fur G-DGRARYEEAP-SEHHDHLIDVNSARVIEFTSPEIEALQREI--ARKHGFRLVGH-RLELYGVPLTSGGDSDDK------------------ 143
B
additional alpha-helix
CxxC motif
C-terminal intrinsically disordered region
C
D
Cys152
Cys114
Cys155
Cys117

Supplemental Figure 4. Structural analysis of BosR reveals non-canonical unique features. A. Multiple sequence alignment (MSA) of BosR and other well-characterized Fur family members. Secondary structure predictions for BosR, based on model presented in **B**, are shown above the MSA; α-helices, β-strands and intrinsically disordered region (IDR) are shown in blue, red and orange, respectively. Residue numbers correspond to BosR. Amino acids known to be involved in regulatory metal coordination (●) are highlighted yellow, green or gray; position 77 is used to discriminate between PerR (Asp, green) and Fur/Zur/Mur/Nur regulators (Glu, magenta). CxxC motif residues (○) involved in structural metal coordination are highlighted in cyan. Asparagine (N) or arginine (R) residues in blue, located in DNA binding helix H4, can be used to distinguish between PerR and Fur, respectively (43). Uniprot IDs for Furs used in MSA: *Campylobacter jejuni* PerR (Q0PBI7; PDB: 6DK4); *Streptococcus pyogenes* PerR (A0A0H2UT39; PDB: 4I7H); *Bacillus subtilis* PerR (P71086; PDB: 3F8N); *Staphylococcus aureus* PerR (Q2G282); *Leptospira interrogans* PerRA (Q72QS5; PDB:5NL9); *Escherichia coli* Zur (P0AC51, PDB: 4MTD) and Fur (P0A9A9, PDB: 2FU4); *Mycobacterium tuberculosis* Zur (P9WN85, PDB: 2O03); *Streptomyces coelicolor* Zur (Q9L2H5, PDB: 3MWM) and Nur (Q9K4F8, PDB: 3EYY); *Francisella tularensis* Fur (Q5NIN6, PDB: 5NBC); *Vibrio cholerae* (P0C6C8; PDB: 2W57); *Pseudomonas aeruginosa* Fur (Q03456, PDB: 6H1C); *Rhizobium leguminosarum* Mur (O07315, PDB: 5FD6); and *Magnetospirillum gryphiswaldense* Fur (V6F4Q0, PDB: 4RB1). **B.** Structural model for BosR dimer predicted by AlphaFold. The N-terminal DNA binding and the C-terminal dimerization domains are colored in violet and green, respectively. The C-terminal IDR (orange), the unique α-helix in the DNA binding domain (yellow) and the CxxC motif (red circle and sticks) are indicated. Side view of BosR dimer (**C**) and zoomed in view of CxxC motif (**D**) are based on model in **B**. Colors in **C** and **D** are as described for **B**.

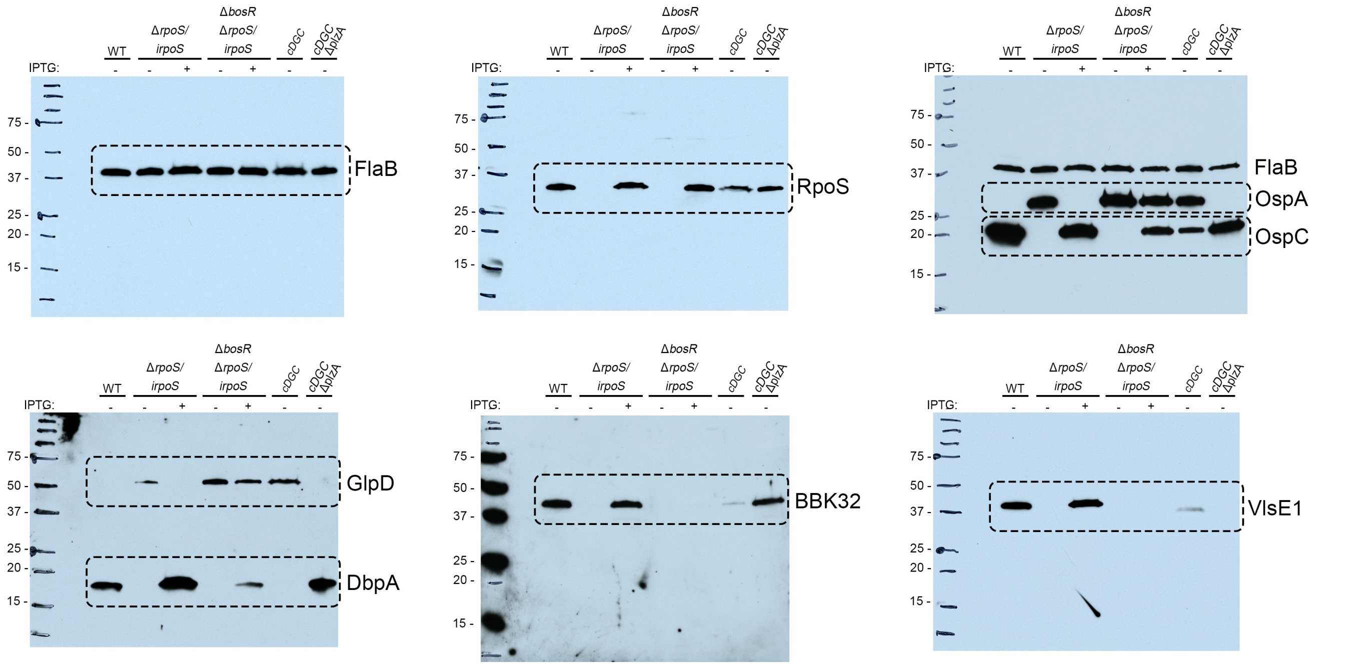

Supplemental Figure 5. Uncropped western blots for Figure 4A. Dotted lines indicate regions that were cropped for the figure. Molecular weight markers (kDa) are shown at the left of each gel. "+" and "–" in all images indicate the presence or absence of IPTG induction.

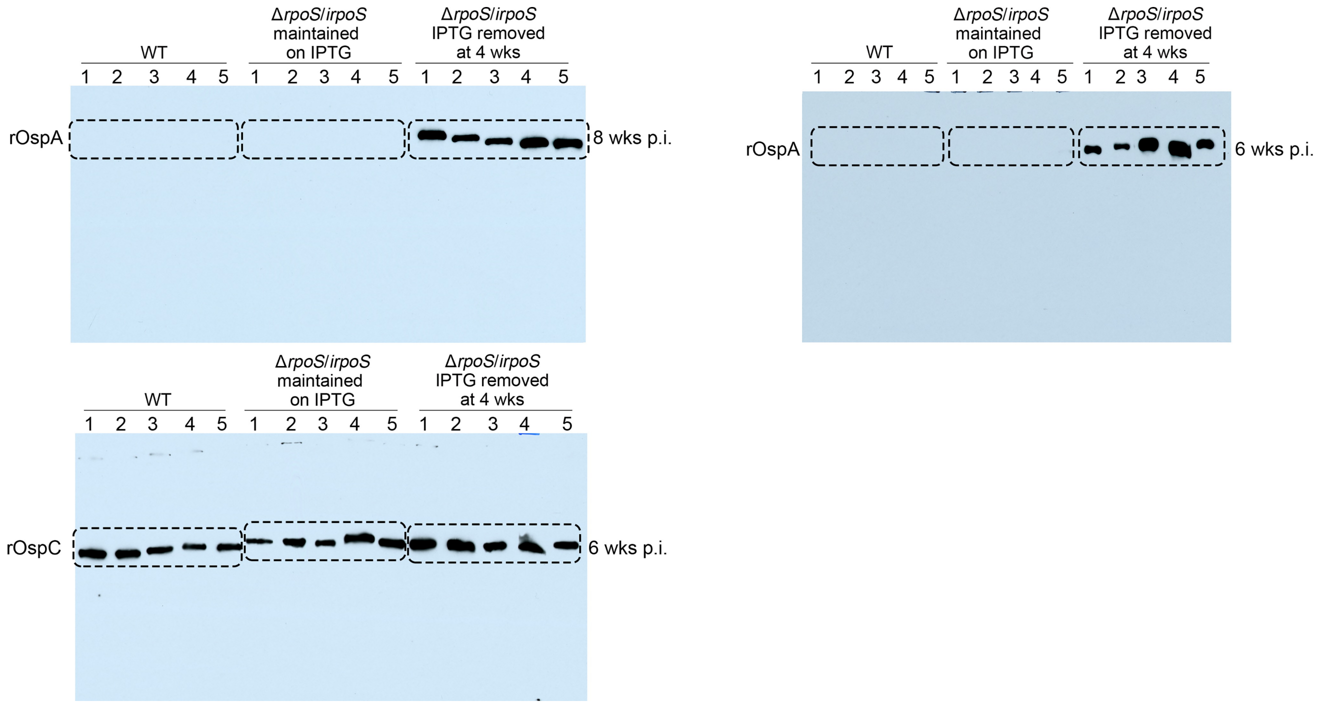

Supplemental Figure 6. Uncropped western blots for Figure 5B. Dotted lines indicate regions that were cropped for the figure.

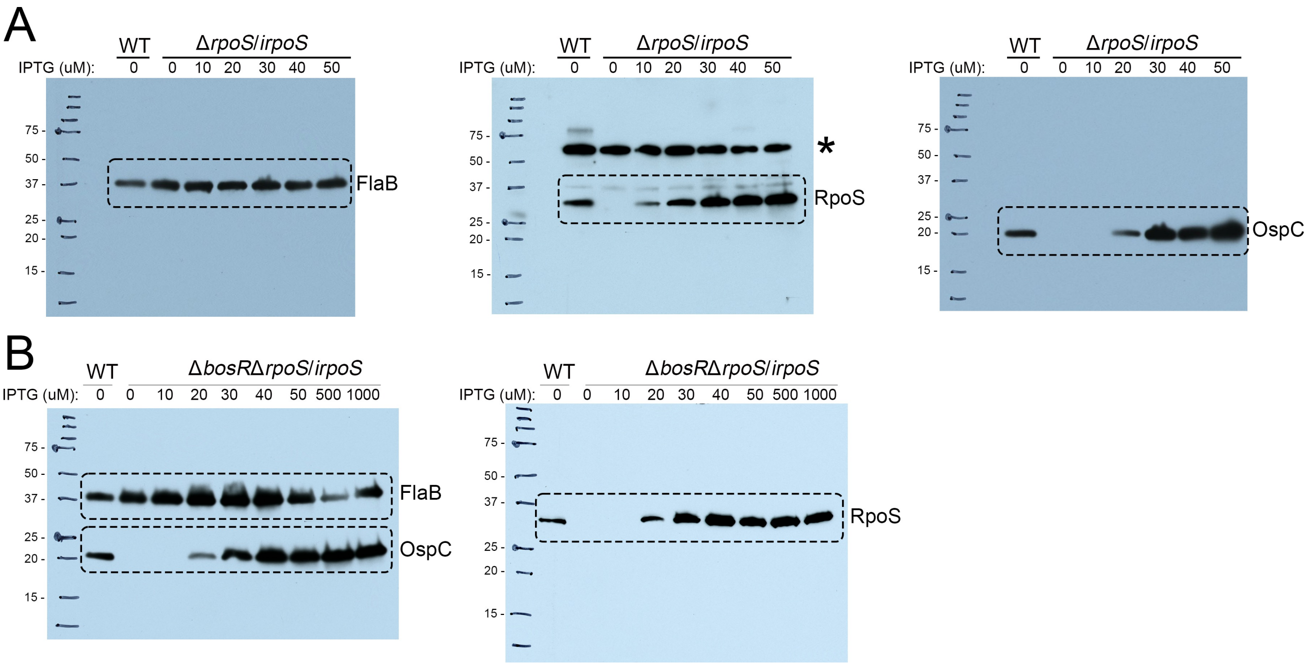

Supplemental Figure 7. Uncropped western blots for Supplemental Figure 2. Dotted lines indicate regions that were cropped for the figure. Molecular weight markers (kDa) are shown at the left of each gel. *, cross-reactive band recognized by rabbit polyclonal anti-RpoS (15), presumably RpoD.

Supplemental References**REFERENCES**

1. Pollack RJ, Telford SR, 3rd, and Spielman A. Standardization of medium for culturing Lyme disease spirochetes. *J Clin Microbiol.* 1993;31(5):1251-5.
2. Groshong AM, Grassmann AA, Luthra A, McLain MA, Provatas AA, Radolf JD, et al. PlzA is a bifunctional c-di-GMP biosensor that promotes tick and mammalian host-adaptation of *Borrelia burgdorferi*. *PLoS Pathog.* 2021;17(7):e1009725.
3. Caimano MJ. Generation of mammalian host-adapted *Borrelia burgdorferi* by cultivation in peritoneal dialysis membrane chamber implantation in rats. *Methods Mol Biol.* 2018;1690:35-45.
4. Akins DR, Bourell KW, Caimano MJ, Norgard MV, and Radolf JD. A new animal model for studying Lyme disease spirochetes in a mammalian host-adapted state. *J Clin Invest.* 1998;101(10):2240-50.
5. Samuels DS. Electrotransformation of the spirochete *Borrelia burgdorferi*. *Methods Mol Biol.* 1995;47:253-9.
6. Groshong AM, Gibbons NE, Yang XF, and Blevins JS. Rrp2, a prokaryotic enhancer-like binding protein, is essential for viability of *Borrelia burgdorferi*. *J Bacteriol.* 2012;194(13):3336-42.
7. Iyer R, Caimano MJ, Luthra A, Axline D, Jr., Corona A, Iacobas DA, et al. Stage-specific global alterations in the transcriptomes of Lyme disease spirochetes during tick feeding and following mammalian host adaptation. *Mol Microbiol.* 2015;95(3):509-38.
8. Caimano MJ, Groshong AM, Belperron A, Mao J, Hawley KL, Luthra A, et al. The RpoS gatekeeper in *Borrelia burgdorferi*: an invariant regulatory scheme that promotes spirochete persistence in reservoir hosts and niche diversity. *Front Microbiol.* 2019;10:1923.
9. Ouyang Z, Kumar M, Kariu T, Haq S, Goldberg M, Pal U, et al. BosR (BB0647) governs virulence expression in *Borrelia burgdorferi*. *Mol Microbiol.* 2009;74(6):1331-43.
10. Stewart PE, Thalken R, Bono JL, and Rosa P. Isolation of a circular plasmid region sufficient for autonomous replication and transformation of infectious *Borrelia burgdorferi*. *Mol Microbiol.* 2001;39(3):714-21.
11. Caimano MJ, Iyer R, Eggers CH, Gonzalez C, Morton EA, Gilbert MA, et al. Analysis of the RpoS regulon in *Borrelia burgdorferi* in response to mammalian host signals provides insight into RpoS function during the enzootic cycle. *Mol Microbiol.* 2007;65(5):1193-217.
12. Caimano MJ, Eggers CH, Gonzalez CA, and Radolf JD. Alternate sigma factor RpoS is required for the in vivo-specific repression of *Borrelia burgdorferi* plasmid lp54-borne *ospA* and *lp6.6* genes. *J Bacteriol.* 2005;187(22):7845-52.
13. Hagman KE, Lahdenne P, Popova TG, Porcella SF, Akins DR, Radolf JD, et al. Decorin-binding protein of *Borrelia burgdorferi* is encoded within a two-gene operon and is protective in the murine model of Lyme borreliosis. *Infect Immun.* 1998;66(6):2674-83.
14. Pappas CJ, Iyer R, Petzke MM, Caimano MJ, Radolf JD, and Schwartz I. *Borrelia burgdorferi* requires glycerol for maximum fitness during the tick phase of the enzootic cycle. *PLoS Pathogens.* 2011;7(7):e1002102.
15. Hyde JA, Trzeciakowski JP, and Skare JT. *Borrelia burgdorferi* alters its gene expression and antigenic profile in response to CO_2 levels. *J Bacteriol.* 2007;189(2):437-45.
16. Xie J, Zhi H, Garrigues RJ, Keightley A, Garcia BL, and Skare JT. Structural determination of the complement inhibitory domain of *Borrelia burgdorferi* BBK32 provides insight into classical pathway complement evasion by Lyme disease spirochetes. *PLoS Pathog.* 2019;15(3):e1007659.
17. Liang FT, Steere AC, Marques AR, Johnson BJ, Miller JN, and Philipp MT. Sensitive and specific serodiagnosis of Lyme disease by enzyme-linked immunosorbent assay with a peptide based on an immunodominant conserved region of *Borrelia burgdorferi vlsE*. *J Clin Microbiol.* 1999;37(12):3990-6.
18. Grassmann AA, Zavala-Alvarado C, Bettin EB, Picardeau M, Benaroudj N, and Caimano MJ. The FUR-like regulators PerRA and PerRB integrate a complex regulatory network that promotes

mammalian host-adaptation and virulence of *Leptospira interrogans*. *PLoS Pathog.* 2021;17(12):e1009078.

19. Joshi NA, and Fass JN. Sickle: A sliding-window, adaptive, quality-based trimming tool for FastQ files. https://github.com/najoshi/sickle. 2022.
20. Magoc T, Wood D, and Salzberg SL. EDGE-pro: Estimated degree of gene expression in prokaryotic genomes. *Evol Bioinform Online.* 2013;9:127-36.
21. Fraser CM, Casjens S, Huang WM, Sutton GG, Clayton R, Lathigra R, et al. Genomic sequence of a Lyme disease spirochaete, *Borrelia burgdorferi*. *Nature.* 1997;390(6660):580-6.
22. Casjens S, Palmer N, van Vugt R, Huang WM, Stevenson B, Rosa P, et al. A bacterial genome in flux: the twelve linear and nine circular extrachromosomal DNAs in an infectious isolate of the Lyme disease spirochete *Borrelia burgdorferi*. *Mol Microbiol.* 2000;35(3):490-516.
23. Love MI, Huber W, and Anders S. Moderated estimation of fold change and dispersion for RNA-seq data with DESeq2. *Genome Biol.* 2014;15(12):550.
24. Team RS. RStudio: Integrated Development for R. http://www.rstudio.com/. 2022.
25. Wagner GP, Kin K, and Lynch VJ. Measurement of mRNA abundance using RNA-seq data: RPKM measure is inconsistent among samples. *Theory Biosci.* 2012;131(4):281-5.
26. Mulay VB, Caimano MJ, Iyer R, Dunham-Ems S, Liveris D, Petzke MM, et al. *Borrelia burgdorferi bba74* is expressed exclusively during tick feeding and is regulated by both arthropod- and mammalian host-specific signals. *J Bacteriol.* 2009;191(8):2783-94.
27. Marchler-Bauer A, Zheng C, Chitsaz F, Derbyshire MK, Geer LY, Geer RC, et al. CDD: conserved domains and protein three-dimensional structure. *Nucleic Acids Res.* 2013;41(Database issue):D348-52.
28. UniProt C. UniProt: a worldwide hub of protein knowledge. *Nucleic Acids Res.* 2019;47(D1):D506-D15.
29. Mitchell AL, Attwood TK, Babbitt PC, Blum M, Bork P, Bridge A, et al. InterPro in 2019: improving coverage, classification and access to protein sequence annotations. *Nucleic Acids Res.* 2019;47(D1):D351-D60.
30. Savojardo C, Martelli PL, Fariselli P, and Casadio R. DeepSig: deep learning improves signal peptide detection in proteins. *Bioinformatics.* 2018;34(10):1690-6.
31. Setubal JC, Reis M, Matsunaga J, and Haake DA. Lipoprotein computational prediction in spirochaetal genomes. *Microbiology.* 2006;152(Pt 1):113-21.
32. Teufel F, Almagro Armenteros JJ, Johansen AR, Gislason MH, Pihl SI, Tsirigos KD, et al. SignalP 6.0 predicts all five types of signal peptides using protein language models. *Nat Biotechnol.* 2022.
33. Kenedy MR, Scott EJ, 2nd, Shrestha B, Anand A, Iqbal H, Radolf JD, et al. Consensus computational network analysis for identifying candidate outer membrane proteins from *Borrelia* spirochetes. *BMC Microbiol.* 2016;16(1):141.
34. Sievers F, Wilm A, Dineen D, Gibson TJ, Karplus K, Li W, et al. Fast, scalable generation of high-quality protein multiple sequence alignments using Clustal Omega. *Mol Syst Biol.* 2011;7:539.
35. Katoh K, Rozewicki J, and Yamada KD. MAFFT online service: multiple sequence alignment, interactive sequence choice and visualization. *Brief Bioinform.* 2019;20(4):1160-6.
36. Jumper J, Evans R, Pritzel A, Green T, Figurnov M, Ronneberger O, et al. Highly accurate protein structure prediction with AlphaFold. *Nature.* 2021;596(7873):583-9.
37. Varadi M, Anyango S, Deshpande M, Nair S, Natassia C, Yordanova G, et al. AlphaFold Protein Structure Database: massively expanding the structural coverage of protein-sequence space with high-accuracy models. *Nucleic Acids Res.* 2022;50(D1):D439-D44.
38. Jones DT, and Cozzetto D. DISOPRED3: precise disordered region predictions with annotated protein-binding activity. *Bioinformatics.* 2015;31(6):857-63.
39. Rego RO, Bestor A, and Rosa PA. Defining the plasmid-borne restriction-modification systems of the Lyme disease spirochete *Borrelia burgdorferi*. *J Bacteriol.* 2011;193(5):1161-71.

40. Pitzer JE, Sultan SZ, Hayakawa Y, Hobbs G, Miller MR, and Motaleb MA. Analysis of the *Borrelia burgdorferi* cyclic-di-GMP-binding protein PlzA reveals a role in motility and virulence. *Infect Immun.* 2011;79(5):1815-25.
41. Pal U, Li X, Wang T, Montgomery RR, Ramamoorthi N, Desilva AM, et al. TROSPA, an *Ixodes scapularis* receptor for *Borrelia burgdorferi*. *Cell.* 2004;119(4):457-68.
42. Frank KL, Bundle SF, Kresge ME, Eggers CH, and Samuels DS. *aadA* confers streptomycin resistance in *Borrelia burgdorferi*. *J Bacteriol.* 2003;185(22):6723-7.
43. Caux-Thang C, Parent A, Sethu R, Maiga A, Blondin G, Latour JM, et al. Single asparagine to arginine mutation allows PerR to switch from PerR box to Fur box. *ACS Chem Biol.* 2015;10(3):682-6.
